# Biomarker Analysis from Patients with Metastatic PDAC Treated with TGFβ Antibody NIS793 plus Abraxane + Gemcitabine versus Abraxane + Gemcitabine Alone in a Phase II, Open-Label, Randomized Study

**DOI:** 10.1158/1078-0432.CCR-26-0805

**Published:** 2026-06-25

**Authors:** Marc Pelletier, Jie Yang, Mukta Joshi, Angelo Grauel, Cara Abecunas, Maria Athina Altzerinakou, Zheng Zhou, Xiaoping Zhu, Kirk Clark, Li Li, Li-Yuan Bai, Michele Milella, Teresa Macarulla Mercade, Wai Meng David Tai, Bruno Bockorny, Peter Grell, Shivan Sivakumar, Mark Ka Wong, Cheng Ean Chee, Thomas Aparicio, Gerald Prager, Geraldine Bostel, Brigitte Schiessl, Heiko Maacke, Glenn Dranoff, Alice Shaw, Claire Fabre, Jennifer Mataraza, Shiva Malek, Viviana Cremasco

**Affiliations:** 1 Novartis BioMedical Research, Cambridge, Massachusetts.; 2 Novartis BioMedical Research, Basel, Switzerland.; 3 Novartis BioMedical Research, East Hanover, New Jersey.; 4 Division of Hematology and Oncology, Clinical Trial Center, China Medical University Hospital, Taichung, Taiwan.; 5 Department of Oncology, University of Verona, Verona, Italy.; 6 Gastrointestinal Cancer Program, Vall d’Hebron University Hospital and Vall d’Hebron Institute of Oncology (VHIO), Barcelona, Spain.; 7 National Cancer Centre Singapore, Duke-NUS Medical School, National University Cancer Institute, Singapore (NCIS), National University Health System, Yong Loo Lin School of Medicine, National University of Singapore, Singapore, Singapore.; 8 Department of Medicine, Harvard Medical School, Beth Israel Deaconess Medical Center, Boston, Massachusetts.; 9 Masaryk Memorial Cancer Institute, Brno, Czech Republic.; 10 Department of Immunology and Immunotherapy, School of Infection, Inflammation and Immunology, College of Medicine and Health, University of Birmingham, Birmingham, United Kingdom.; 11 Westmead Hospital, Westmead, Australia.; 12 Department of Haematology – Oncology, National University Cancer Institute, Singapore, Singapore.; 13 Hôpital Saint-Louis/Assistance Publique–Hôpitaux de Paris (AP-HP), Paris, France.; 14 Department of Medicine I, Division of Oncology, Medical University of Vienna, Vienna, Austria.

## Abstract

**Purpose:**

Transforming growth factor β (TGFβ) plays a dual role in cancer, acting as a tumor suppressor early in the disease but promoting progression and immune evasion when dysregulated. In pancreatic ductal adenocarcinoma (PDAC), TGFβ-driven desmoplasia fosters chemoresistance and immunosuppression, limiting therapeutic efficacy. NIS793, a fully human mAb targeting TGFβ, demonstrated antifibrotic and immunomodulatory activity in preclinical models and early-phase trials.

**Patients and Methods:**

We conducted a randomized, open-label, phase II study in treatment-naïve patients with metastatic PDAC (mPDAC) to evaluate NIS793 ± spartalizumab (anti–PD-1) combined with nab-paclitaxel (or Abraxane)/gemcitabine (ABRA/GEM) versus ABRA/GEM alone. The primary endpoint was progression-free survival (PFS); secondary endpoints included overall survival (OS), safety, pharmacokinetics, and biomarker analyses. Exploratory assessments included paired tumor RNA sequencing, cell-free DNA profiling, and plasma proteomics.

**Results:**

NIS793 demonstrated target engagement and suppression of TGFβ signaling, confirmed by transcriptomic and proteomic analyses. Stromal remodeling was evident, with significant downregulation of cancer-associated fibroblast markers (*Acta2*, *Fap*) and collagen-related signatures. Despite proof of mechanism, clinical efficacy was not observed: Median PFS and OS were comparable or numerically worse in the NIS793 arm versus control (HR for OS in NIS793 + ABRA/GEM vs. ABRA/GEM: 1.32; 95% confidence interval, 0.84–2.07). The safety profile was manageable, with no unexpected toxicities. Biomarker data revealed increased expression of neutrophil-related genes after treatment, suggesting potential induction of tumor-promoting inflammation.

**Conclusions:**

NIS793 effectively inhibited TGFβ signaling and led to stromal remodeling but failed to improve outcomes in mPDAC. These findings highlight the complexity of TGFβ biology and caution against its blockade in combination with chemotherapy for PDAC. Future strategies should consider context-dependent effects of TGFβ inhibition.

**Translational Relevance**

Transforming growth factor β (TGFβ) is known to promote tissue and microenvironment dysfunction that collectively promote tumor progression. Previous preclinical studies suggested that blocking TGFβ could remodel the tumor microenvironment, reduce fibrosis, and enhance responses to checkpoint inhibitors and cytotoxic agents.

This randomized phase II trial showed that NIS793, a mAb targeting TGFβ, effectively inhibited TGFβ signaling and reduced stromal activation in metastatic pancreatic ductal adenocarcinoma (mPDAC) but failed to provide clinical benefits, potentially because of broader effects of TGFβ in other compartments and promotion of tumor-supportive inflammation.

This study highlights the complexity of TGFβ biology and the need for deeper mechanistic studies to understand context-dependent effects of TGFβ inhibition, particularly its impact on innate immunity and tumor-promoting inflammation. It also suggests caution for clinical practice in combining TGFβ inhibitors with chemotherapy for PDAC, underscoring the importance of biomarker-driven patient selection and rational combination strategies.

## Introduction

Transforming growth factor β (TGFβ) is part of a large family of structurally related cytokines that includes bone morphogenetic proteins, growth and differentiation factors, activins, and inhibins. In mammals, three isoforms of the TGFβ ligand—TGFβ1, TGFβ2, and TGFβ3—are expressed. Under physiologic conditions, TGFβ plays a crucial role in maintaining cellular homeostasis by limiting the proliferation of epithelial, endothelial, neuronal, and hematopoietic cells through antiproliferative and apoptotic mechanisms (1–3). Dysregulation of this pathway has been implicated in a wide range of human diseases, including cardiovascular disorders, fibrosis, reproductive abnormalities, impaired wound healing, and various cancers (1, 4).

In cancer biology, TGFβ has a dual role. Initially recognized for its tumor-suppressive properties due to its cytostatic and apoptotic effects, TGFβ can paradoxically promote tumor progression when its signaling is altered (1, 5). Somatic mutations in components of the TGFβ pathway, such as the frequent deletion of Smad4 in pancreatic ductal adenocarcinoma (PDAC), allow cancer cells to evade growth inhibition while enhancing their invasive and metastatic potential (6–8).

Beyond its direct effects on tumor cells, TGFβ also shapes the tumor microenvironment (TME) by promoting angiogenesis, immune evasion, and the activation of cancer-associated fibroblasts (3, 4, 9). This leads to a desmoplastic reaction characterized by excessive extracellular matrix (ECM) deposition and tissue stiffening, which has been suggested to contribute to resistance against chemotherapies and immunotherapeutic agents (6–8). This phenomenon is quite prominent in tumor types like PDAC, in which activated stellate cells, the primary drivers of the desmoplastic reaction in the pancreas, further reinforce a chemoprotective microenvironment by scavenging therapeutic agents and secreting factors that help tumor cells resist cytotoxic and cytostatic treatments (9). Moreover, the fibrotic stroma contributes to immunosuppression through the promotion of an immune-excluded environment, as well as by way of the secretion of molecules such as hyaluronic acid, thrombospondin, and tenascin C that directly halt T-cell function. In line with this concept, clinical studies in metastatic urothelial carcinoma have linked TGFβ-induced fibroblast activation with poor responses to immune checkpoint inhibitors like atezolizumab and nivolumab (10).

NIS793 is a fully human IgG2 mAb that binds TGFβ1 and TGFβ2 with high affinity and TGFβ3 with lower affinity. It cross-reacts with rodent and cynomolgus monkey TGFβ and demonstrates blocking activity in various *in vitro* and *in vivo* assays. NIS793 has shown potent neutralizing activity against TGFβ1 and TGFβ2, with limited effect on TGFβ3 (11). In preclinical models of cancer, NIS793 significantly reduced the accumulation of myofibroblasts, halting intratumoral fibrosis development and ultimately enhancing the efficacy of anti–PD-1 therapy in immunocompetent mice (12). Additionally, NIS793 improved the activity of gemcitabine/5-fluorouracil (5-FU)–based chemotherapy in pancreatic cancer models (13). In a phase Ib trial (NCT02947165), NIS793, alone or with spartalizumab, a PD-1 blocking antibody, showed a manageable safety profile and preliminary signs of clinical activity, including partial responses and prolonged stable disease in a subset of patients (11).

In light of the encouraging results from the phase Ib trial with NIS793 and based on the mechanistic hypothesis that TGFβ blockade may alleviate some of the structural aberrations of the TME and promote better chemo/immune therapy activity, NIS793 was tested in patients with metastatic PDAC (mPDAC), a tumor type characterized by extensive tissue derangements and desmoplasia. This study was a two-part phase II study to assess the efficacy and safety of NIS793 with and without spartalizumab in combination with nab-paclitaxel (or Abraxane)/gemcitabine (ABRA/GEM) versus ABRA/GEM in untreated mPDAC. The current article presents data from this phase II study, focusing on biomarker analyses, together with preclinical studies to complement the observed clinical findings.

## Patients and Methods

### Study design

This phase II, randomized, open-label, multicenter study evaluated the safety and efficacy of NIS793, with or without spartalizumab, in combination with ABRA/GEM versus ABRA/GEM alone in treatment-naïve patients with mPDAC. The study was conducted in two parts: a safety run-in and a randomized treatment part. The safety run-in assessed the tolerability of NIS793 + spartalizumab + ABRA/GEM, enrolling at least six patients. Following safety confirmation, 153 patients were randomized equally into three arms: arm 1 (NIS793 with spartalizumab and ABRA/GEM), arm 2 (NIS793 with ABRA/GEM), and arm 3 (ABRA/GEM). Eligible patients were adults with histologically or cytologically confirmed, treatment-naïve mPDAC and measurable disease per RECIST 1.1. Inclusion required an Eastern Cooperative Oncology Group (ECOG) performance status ≤1 and willingness to undergo tumor biopsies at screening and during treatment (Supplementary Fig. S1). The study followed International Council for Harmonisation of Technical Requirements for Registration of Pharmaceuticals for Human Use E6 Good Clinical Practice guidelines based on the Declaration of Helsinki. The study protocol and all amendments were reviewed by the Independent Ethics Committee or Institutional Review Board for each center. Informed consent was obtained from each participant in writing before screening and before any study-specific procedure was performed.

The primary objective of the safety run-in phase was to evaluate the safety and tolerability of combining NIS793 and spartalizumab with ABRA/GEM. Key endpoints included the incidence of dose-limiting toxicities (DLT) within the first 4 weeks, treatment-emergent adverse events (AE), and serious AEs (SAE), as well as changes in laboratory values, vital signs, and ECGs. Tolerability was further assessed through dose modifications and intensity. In the randomized part, the primary objective was to assess progression-free survival (PFS) for two experimental arms—NIS793 with spartalizumab and chemotherapy, and NIS793 with chemotherapy—compared with chemotherapy alone. Secondary objectives included evaluating overall survival (OS), safety and tolerability, preliminary antitumor activity, CD8 and PD-L1 status, immunogenicity, and pharmacokinetics (PK) of the study drugs across treatment arms. Exploratory readouts of target engagement and proof of mechanism (POM) were assessed using bulk RNA sequencing (RNA-seq) and SomaLogic analyses.

### Study treatment

The treatment regimen included NIS793 available in two formulations: a 100 mg lyophilized powder for solution for infusion and a 700 mg/7 mL (100 mg/mL) liquid concentrate for solution infusion. Spartalizumab (PDR001) was provided as a 100 mg/4 mL liquid concentrate for solution infusion. Additionally, gemcitabine (1,000 mg/m^2^ on days 1, 8, and 15) and nab-paclitaxel (125 mg/m^2^ on days 1, 8, and 15) were included in the regimen as locally approved and available chemotherapeutic agents. The study treatment was administered as a 28-day treatment cycle. NIS793 was administered at 2,100 mg every 2 weeks, spartalizumab at 400 mg every 4 weeks, and ABRA/GEM as per standard dosing.

### Cell-free DNA extraction processing and data analysis

Baseline cell-free DNA (cfDNA) samples were collected from enrolled patients for quantification of circulating tumor DNA (ctDNA) levels and baseline genomic profiling. The PanCancer cfDNA assay and panel version 2.1 from Novartis BioMedical Research Next Generation Diagnostics laboratory were used to generate data. cfDNA was extracted from approximately 5 mL of plasma (QIAamp Circulating Nucleic Acid Kit, QIAGEN) per the manufacturer’s instructions and then constructed into sequencing libraries with end repair, A-tailing, unique molecular identifier (UMI)–containing adaptor ligation, and PCR amplification using the TruSeq Nano Library Preparation Kit (Illumina). The constructed cfDNA libraries were hybridized to RNA baits (SureSelect, Agilent) targeting 579 cancer-related genes. The captured library was sequenced to target a median of 300 million sequencing reads per library. Sequencing data were aligned to the reference human genome (build hg38) using the Burrows-Wheeler Aligner (BWA-MEM; arXiv 1303.3997), followed by the application of UMI-based consensus building and error suppression using the fgbio tools (fgbio.com). Then, the Genome Analysis Toolkit was used for local realignment and base quality score recalibration (14, 15). The final output consisted of an error-suppressed, unique set of aligned reads suitable for variant calling and further analysis. Single- and multi-nucleotide variants were identified with MuTect (RRID:SCR_000559; ref. 16) and short insertion/deletion events using Pindel (RRID:SCR_000560; ref. 17). Focal and arm-level copy-number events and tumor mutation burden were called using PureCN (18), whereas structural variants, including chromosomal rearrangements, were called using Socrates (19). Artifacts and germline variants were filtered out using a pool of normal samples and databases such as the Single Nucleotide Polymorphism Database (dbSNP; RRID:SCR_002338) and gnomAD, respectively. Annotation of called variants was performed using dbSNP and the Catalogue of Somatic Mutations in Cancer (RRID:SCR_002260) databases, among others.

### Bulk RNA-seq sample processing and data analysis

Paired tumor biopsies (screening and C3D2-4) were obtained for RNA-seq analysis. Total RNA was extracted from a biopsy specimen using an extraction kit compatible with formalin-fixed, paraffin-embedded material. Extracted RNA was assayed for quantity (RiboGreen) and RNA fragment length [Agilent TapeStation (RRID:SCR_019547)]. Ribosomal RNA (rRNA) from extracted total RNA was depleted using the RNase H approach. The rRNA-depleted sample was then fragmented, converted to cDNA, and carried through the remaining steps of next-generation sequencing library construction: end repair, A-tailing, indexed adaptor ligation, and PCR amplification using the TruSeq RNA v2 Library Preparation kit (Illumina). The captured library was sequenced to target a median of 50 million sequencing reads per library. Sequence data were aligned to the reference human genome (build hg19) using STAR (RRID:SCR_004463; ref. 20). Mapped reads were assembled into transcripts, and transcript abundances were estimated and normalized using Cufflinks (RRID:SCR_014597; ref. 21). Finally, HTSeq (RRID:SCR_005514; ref. 22) was used to count the number of reads mapping to each gene. Data normalization for differential expression analysis was carried out using the edgeR package (RRID:SCR_012802; ref. 23). Gene set enrichment analysis (GSEA; RRID:SCR_00319; R package fgsea, v1.26.0) was performed using relevant gene signature databases to explore pathway-level alterations, and enrichment scores were visualized to facilitate the comparison of functional changes across arms. All statistical and computational analyses were performed in R 3.6.1.

### SomaScan analysis

The proteomic profiling of patient plasma employed the SomaScan Assay version 5 platform, which quantifies protein abundance by measuring relative fluorescence units (RFU) of modified aptamers (SOMAmer). These RFU values reflect protein antigenicity and can be interpreted as changes in protein abundance. Both raw and normalized data were analyzed, with normalization techniques including median normalization, quantile normalization, and proprietary methods by SomaLogic. Data analysis utilized open-source microarray software from the R/Bioconductor consortium, including the arrayQualityMetrics package (RID:SCR_001335) for quality assessment. Mean SOMAmer expression scores were calculated based on gene signatures for Reactome Collagen Formation and an internally derived TGFβ signature. Statistical analysis involved paired *t* tests comparing cycle 1 day 1 with subsequent time points.

### Preclinical toxicology studies

Based on cross-species sequence homology, affinity, and binding of NIS793 to rat, cynomolgus monkey, and human TGFβ, as well as *in vitro* and *in vivo* data demonstrating the pharmacologic activity of NIS793 on proliferation, cytokine release, and antitumor efficacy (11, 12), rat and cynomolgus monkey were selected as pharmacologically relevant species for the nonclinical safety evaluation of NIS793.

The nonclinical safety program conducted with NIS793 to date comprises repeat-dose toxicology studies in rats for up to 13 weeks and in cynomolgus monkeys for up to 8 weeks, as well as a tissue cross-reactivity study using human and monkey tissues. Two pivotal good laboratory practice (GLP) animal studies were performed: a 13-week study in rats with a 4-week recovery period using weekly intravenous doses of 1, 3, and 10 mg/kg/week (Study No.: XMA-014-13) and a 4-week study in cynomolgus monkeys with a 4-week recovery period at weekly dosing of 2, 8, and 16 mg/kg/week (Study No.: 1570330). Additionally, an 8-week exploratory dose-range finding study in male rats (at intravenous doses of 0, 30, and 90 mg/kg, once every 10 days) and an 8-week exploratory dose-range finding study in male monkeys [at intravenous doses of 0, 30/90 mg/kg (30 on days 1, 11, and 21 followed by 90 on days 31, 41, and 51), and 65 mg/kg (once every 10 days)] were conducted. In the tissue reactivity study (Study No. 1570329a), the binding of NIS793 to a full panel of 42 different tissues from humans and cynomolgus monkeys was tested. All principal toxicology studies (13-week rat, 4-week monkey, and tissue cross-reactivity study) were performed in a test facility that is part of the national GLP monitoring program of an EU member state, Organisation for Economic Co-operation and Development member country, or fully adherent to the Mutual Acceptance of Data (MAD) principles and found to be in compliance with GLP. The studies were contracted by Xoma or Novartis at contract research organizations.

### Crystallography and structure determination

TGFβ1 and a slight excess of NIS793 Fab were complexed and incubated at 4°C. To remove excess Fab, the sample was purified on a Superdex-200 (16/600) column equilibrated with 25 mmol/L HEPES (pH 7.4) and 50 mmol/L NaCl. Fractions were analyzed by SDS-PAGE. Fab and antigen complexes were pooled for crystallization. The final antigen-Fab complex was concentrated to approximately 8.5 mg/mL. One percent of 1 mg/mL trypsin (v/v) was added to 120 μL of the protein complex (8.5 mg/mL). The samples were dispensed onto 96-well sitting-drop crystallization plates using a Mosquito liquid handler (SPT Labtech). Plates were incubated at 20°C in a Rock Imager incubator (Formulatrix). Diffracting crystals of TGFβ1–NIS793 were obtained under conditions containing 0.05 mol/L ammonium sulfate, 0.05 mol/L Bis-Tris (pH 6.5), and 30% pentaerythritol propoxylate. Crystals were cryo-protected in 10% ethylene glycol and flash-cooled in liquid nitrogen. X-ray diffraction data were collected at beamline 17ID (IMCA-CAT) at the Advanced Photon Source at Argonne National Laboratories (24–27). The dataset was processed using autoPROC and the CCP4 suite. Molecular replacement was performed by PHASER by searching for two copies of TGFβ1 (PDB: 3KFD, chain A) and two copies of the in-house NIS793 Fab structure. The TGFβ1–NIS793 Fab structure was refined and manually built through several rounds using BUSTER (RRID:SCR_015653) and COOT (RRID:SCR_014222). The final model quality was evaluated by MolProbity (RRID:SCR_014226; ref. 28).

## Results

### Lack of clinical benefit from NIS793 combination with ABRA/GEM in mPDAC

This phase II, randomized, open-label, multicenter study evaluated the safety and efficacy of NIS793, with or without spartalizumab, in combination with ABRA/GEM versus ABRA/GEM alone in treatment-naïve patients with mPDAC (Supplementary Fig. S1). Overall, 11 patients were treated in the safety run-in part, and 153 patients were treated in the randomized part (50 in arm 1 for NIS793 + ABRA/GEM + spartalizumab, 51 in arm 2 for NIS793 + ABRA/GEM, and 52 in arm 3 for ABRA/GEM). Baseline demographic and disease characteristics for the study are reported in Supplementary Tables S1 and S2. The disposition of patients in the safety run-in and randomized parts has been summarized in Supplementary Table S3.

Overall, the most common AEs were anemia and nausea, with neutropenia and anemia reported as the most frequent grade ≥3 treatment-related AEs in all three arms (Supplementary Tables S4–S6). Treatment-related AEs were reported in 97.8%, 91.8%, and 84.4% in arm 1 (NIS793 + ABRA/GEM + spartalizumab), arm 2 (NIS793 + ABRA/GEM), and arm 3 (ABRA/GEM). Treatment-related AEs with grade ≥3 were less frequent in arm 3 (53.3%; ABRA/GEM) compared with arm 1 (73.9%; NIS793 + ABRA/GEM + spartalizumab) and arm 2 (75.5%; NIS793 + ABRA/GEM). Fatal SAEs related to treatment were only reported in arm 1 (2.2%). Treatment-related AEs leading to study drug discontinuation were reported in 10.9%, 8.2%, and 6.7% in arms 1, 2, and 3, respectively. In addition, AEs leading to dose adjustment/interruption were reported in 87%, 75.5%, and 73.3% in arms 1, 2, and 3, respectively (Supplementary Table S5). AEs of special interest (AESI) were also reported in both the safety run-in part and the randomized part, with the most prevalent NIS793 AESIs in both parts of the study being infusion-related reactions, skin toxicity, and hemorrhage (Supplementary Table S7). Of note, two on-treatment deaths were reported in the safety run-in portion of the study (one each: study indication and general physical condition abnormal, not related to study treatment) and 15 in the randomized part (seven related to the study indication, three due to sepsis, and one each due to dyspnea, hemorrhagic gastritis, hepatic failure, renal failure, and septic shock; of these, hemorrhagic gastritis was related to study treatment, possibly due to from disease progression and metastasis).

**Figure 1.**
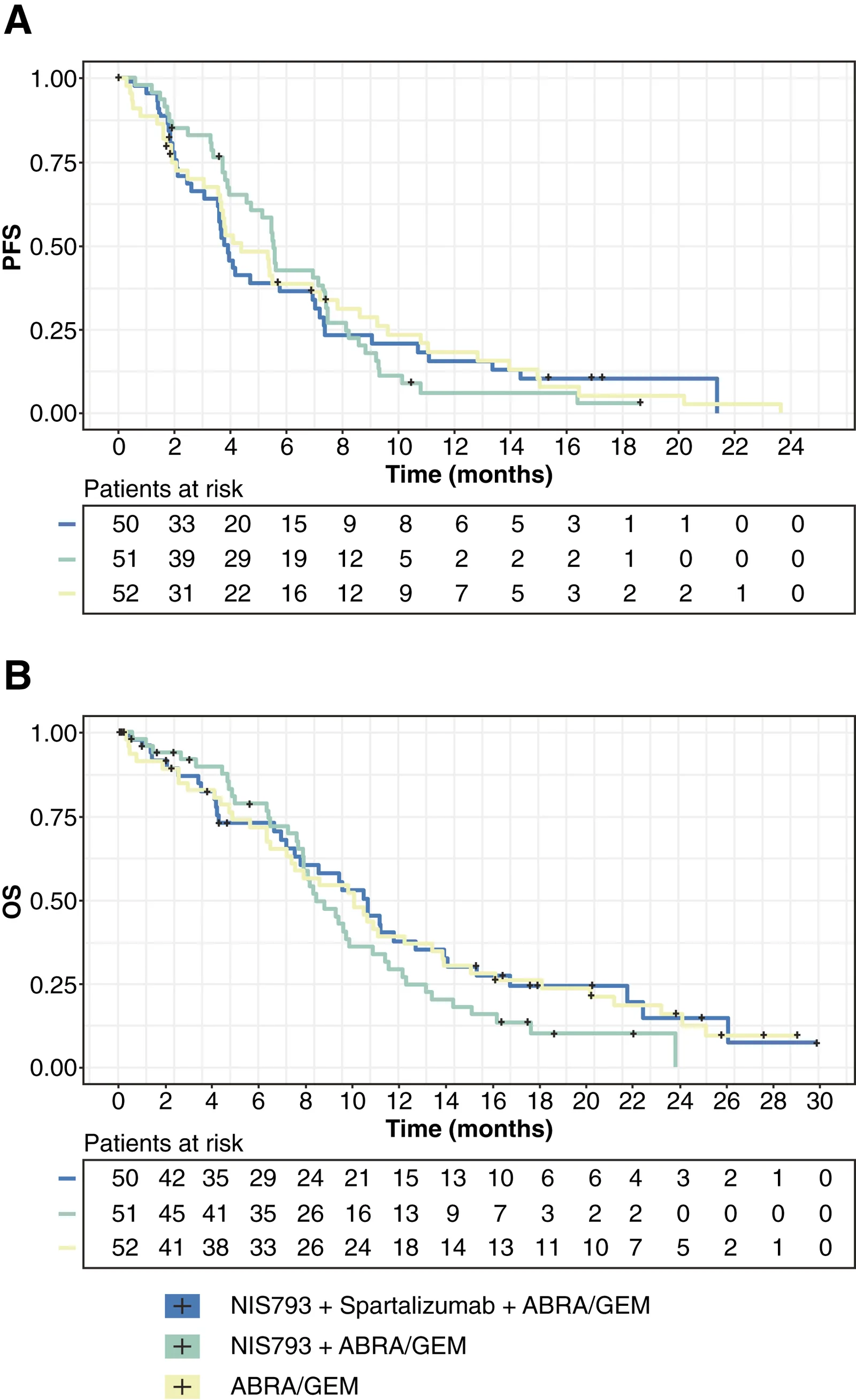
Survival outcome. Kaplan–Meier plot of (**A**) time to PFS per RECIST 1.1 and (**B**) OS.

Analysis of PFS curves showed a lack of benefit in patients in arm 2 (NIS793 + ABRA/GEM) compared with arm 3 [ABRA/GEM; PFS was 5.52 months (95% confidence interval, CI, 3.94–7.29) in arm 2 and 4.37 months (95% CI, 3.55–7.20) in arm 3; HR (one-sided 90% CI) in arm 2 vs. arm 3 was 1.08 (0, 1.44); Fig. 1A; Supplementary Tables S8 and S9]. A similar lack of benefit in PFS extension was also observed for arm 1 [NIS793 + ABRA/GEM + spartalizumab; PFS 3.91 months (95% CI, 3.06–6.93) in arm 1; HR (one-sided 90% CI) in arm 1 versus arm 3 was 1.02 (0, 1.37)]. The median OS estimate was 10.7 months (95% CI, 7.2–12.7) in arm 1 and 10.1 months (95% CI, 6.5–13.4) in arm 3. The HR computed using unadjusted Cox proportional hazard (PH) was 0.97 (95% CI, 0.61–1.53). Overall, 41 (80.4%) participants died in arm 2, and 40 (76.9%) died in arm 3. The median OS was 8.5 months (95% CI, 7.7–9.9) in arm 2 and 10.1 months (95% CI, 6.5–13.4) in arm 3. The median OS confirmed a lack of benefit from NIS793 treatment and instead revealed a trend for decreased survival in patients in arm 2 (NIS793 + ABRA/GEM) compared with arm 1. The HR was 1.32 (95% CI, 0.84–2.07; Fig. 1B; Supplementary Tables S10 and S11). Although not statistically significant, the Kaplan–Meier method estimated the OS rate at month 12 to be 29.2% (95% CI, 16.8–42.8) in arm 2 and 39.1% (95% CI, 25.2–52.8) in arm 3, suggesting a trend for decreased survival in patients receiving NIS793 + ABRA/GEM. The follow-up time for PFS was 5.8 months in the NIS793 group and 6.5 months in the ABRA/GEM group, and the follow-up time for OS was similar between the NIS793 group (12.4 months) and the ABRA/GEM group (12.5 months). Patients progressed as a combination of progression of primary lesions and new metastatic lesions. Notably, emerging data from the parallel phase III pivotal study of NIS793 in combination with ABRA/GEM also revealed an unfavorable benefit–risk profile (ct.gov/NCT04935359, manuscript under consideration), corroborating the trends observed in phase II and leading to program termination.

### Different patient baseline features and underlying mutational features are equally represented in each study arm

**Figure 2.**
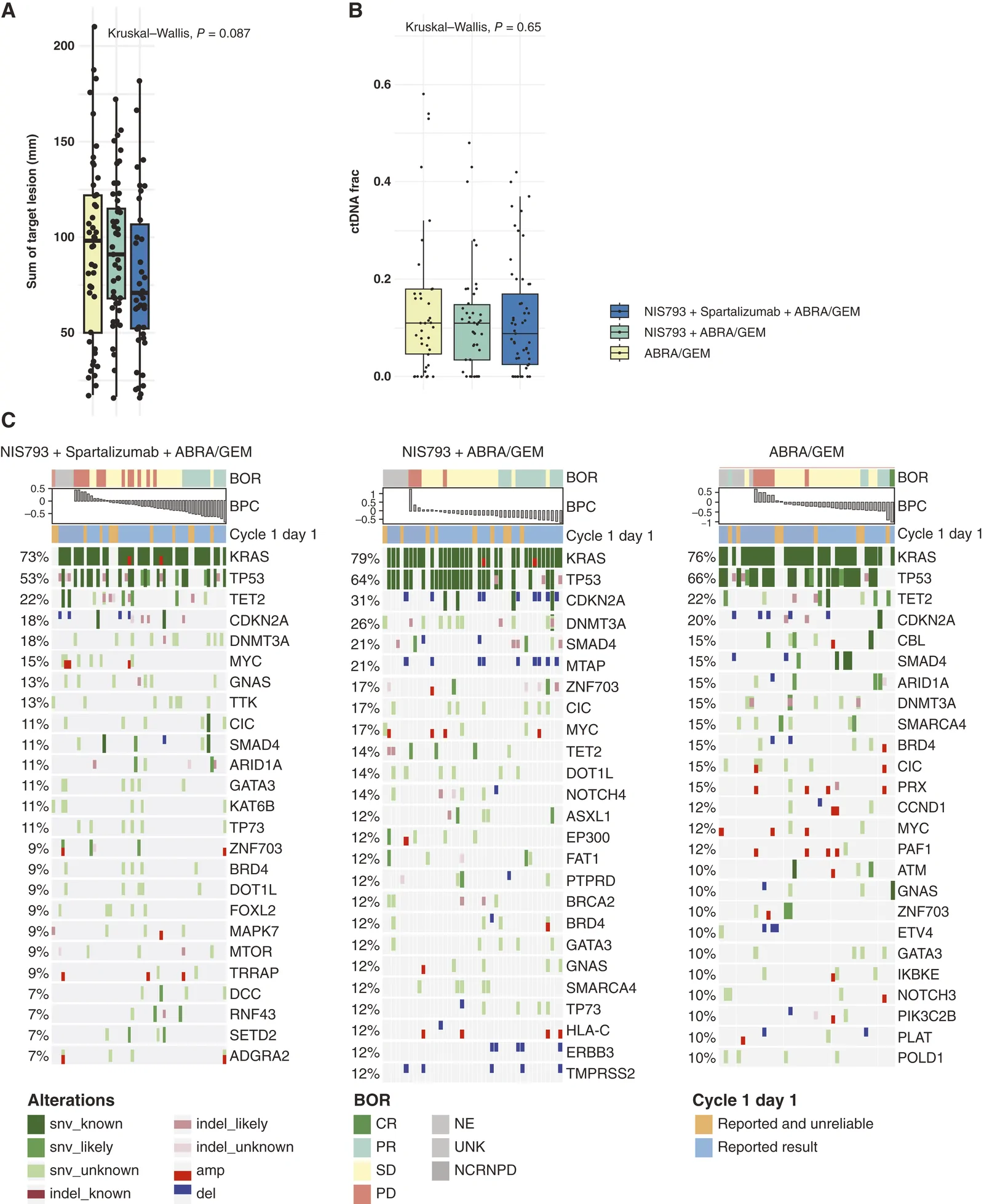
Tumor characteristics at baseline. **A,** Tumor lesion size. **B,** ctDNA fraction. **C,** Oncoprint of tumor genetic alterations across patients and cohorts (arm 1, *n* = 41; arm 2, *n* = 42; arm 3, *n* = 55). BOR, best overall response; BPC, best percent change; CR, complete response; NCRNPD, non complete response/non progression disease; NE, not evaluable; PR, partial response; SD, stable disease; UNK, unknown.

One potential explanation for the lack of efficacy and potentially greater tumor progression observed in arm 2 (NIS793 + ABRA/GEM) could lie in the enrollment of patients with specific baseline or tumor characteristics in that cohort. However, as shown in Supplementary Table S1, patient demographics were similar across the three cohorts, with no differences between age/gender/race of enrolled patients or ECOG performance status. Tumor size at baseline and ctDNA fraction were also similar between groups (Fig. 2A and B). To ascertain whether enrichment for certain mutations that may drive differential response to TGFβ inhibition could be found between groups, we then compared the tumor mutation landscape in ctDNA from the enrolled patients. As shown in Fig. 2C, the mutational makeup of patients was quite diverse, but no remarkable enrichment for any specific trait was observed in any of the treatment arms. Overall, *Kras* and *Tp53* were the most frequent mutations observed at baseline. Mutations in *Smad4*, a common alteration known to affect response to TGFβ signaling in the TME, were also discernible, but no significant difference in their occurrence was noted between the treatment groups (Fig. 2C). Altogether, these findings suggest that clinical observations from the current study are unlikely to stem from unbalanced representation of patients with certain phenotypes or tumor differences at baseline.

### Pharmacologic properties of NIS793

Another hypothesis that could explain the observed clinical outcome is an unforeseen behavior of the NIS793 molecule. However, the PK profile of NIS793 in this study was consistent with that observed in its earlier clinical evaluation (11) and exhibited the typical behavior of a mAb (Supplementary Table S12). In addition, as expected, no drug–drug interaction between NIS793 and ABRA/GEM was seen, nor was immunogenicity observed (no antidrug antibody incidence after NIS793).

**Figure 3.**
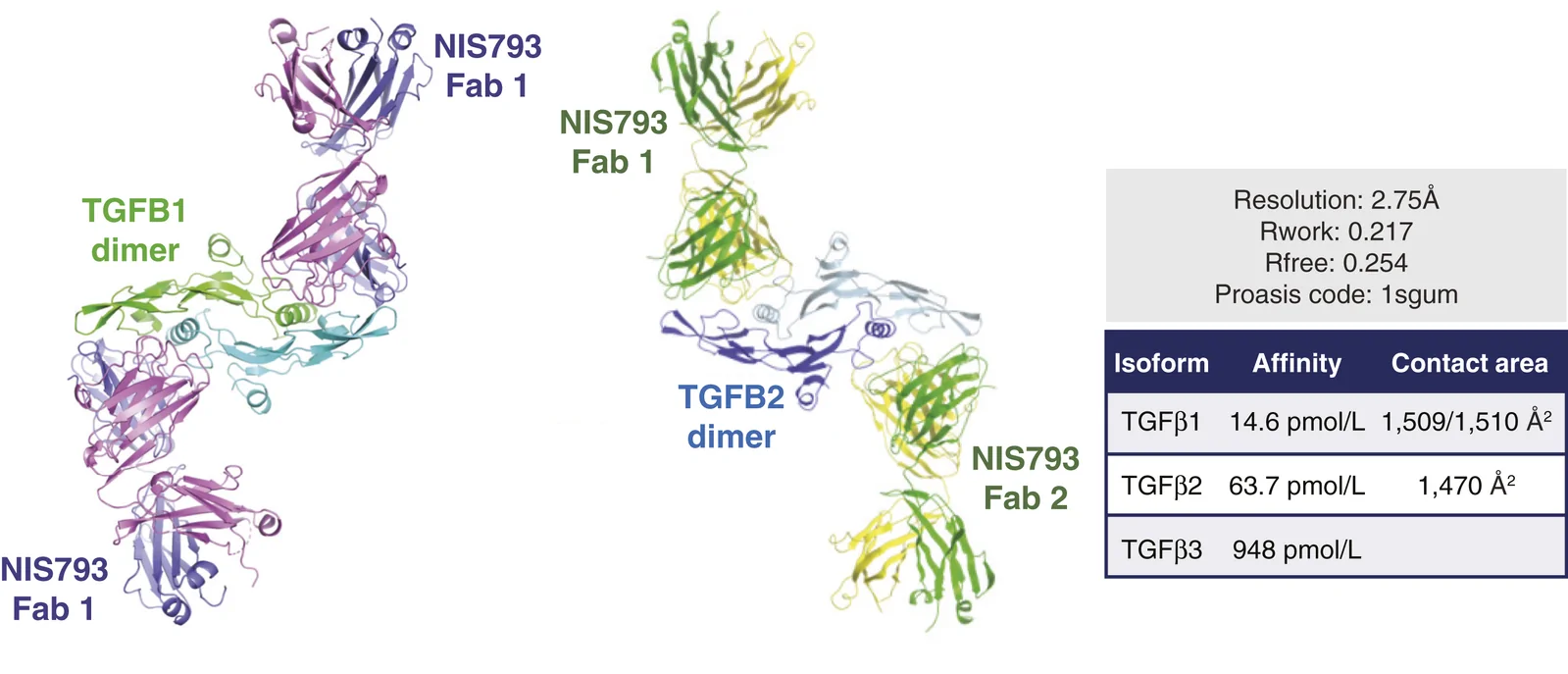
Crystal structure of NIS793. The ribbon diagram depicts the TGFβ1–NIS793 Fab complex structure. The two chains of the TGFβ1 dimer are highlighted in green and cyan, respectively. The heavy chain and light chain of the NIS793 Fab fragment are colored in violet and blue, respectively. The active form of the TGFβ1 dimer is recognized by two NIS793 Fabs. The NIS793 antibody does not disrupt TGFβ1 dimer formation; instead, it blocks the receptor binding site. The conformational epitope includes A279, L280, N292, P327-S331, Q335, K338, V339, L342, Q345, H346 from TGFβ1 chain B, and L306-W310, I366, Y368-R372, E377, L379, S380 from TGFβ1 chain.

In addition to these clinical data, we also reviewed preclinical insights that may suggest atypical pharmacologic properties of NIS793 leading to the unexpected clinical findings. In particular, as TGFβ molecules are known to operate as dimers, one hypothesis that was investigated preclinically was whether NIS793 may induce stoichiometry-driven effects. Crystal structure resolution of the antibody/ligand complex, however, revealed that the NIS793 epitope resides away from the TGFβ dimerization interface (Fig. 3), and it is thereby unlikely to lead to accumulation of monomers (which, in any case, have unclear functional relevance).

Notably, no unexpected liabilities were depicted from preclinical toxicology studies either, as discussed in Supplementary Table S13.

Collectively, these data highlight a normal pharmacologic profile for NIS793 and suggest that the observed clinical effects are not secondary to deviant behaviors of the molecule itself but have instead biological roots.

### Target engagement and modulation by NIS793

In order to gain more insights into the biological activity of NIS793, pharmacodynamic (PD) assessment was carried out through RNA-seq analysis of paired tumor biopsies (screening vs. C3 D2–4). Notably, because of the paucity of paired biopsies collected for RNA-seq assessment from arm 1 and considering the focus of these analyses on deciphering the effects of NIS793, the tissue RNA-seq analyses described below and throughout the article have only been conducted for arm 2 (NIS793 + ABRA/GEM) and arm 3 (ABRA/GEM) of the randomized trial portion.

**Figure 4.**
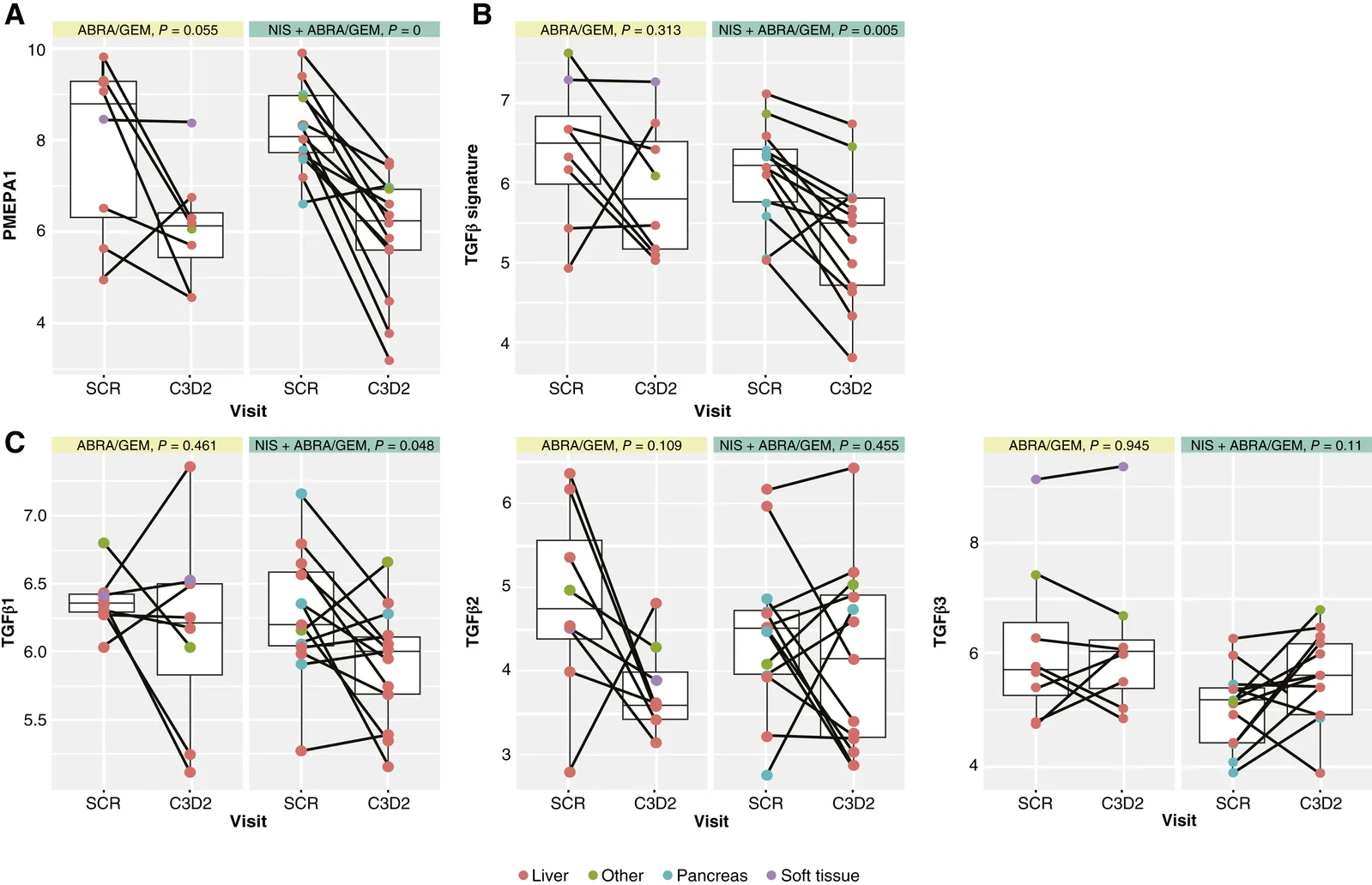
Demonstration of PD from NIS793. Reduction in PMEPA1 expression (**A**) and TGFβ signature (**B**) in arm 2 as detected by RNA-seq from paired biopsies. **C,** TGFβ isoform expression in paired biopsies (RNA-seq). *n* = 8 paired biopsies for ABRA/GEM and 13 paired biopsies for NIS793 + ABRA/GEM.

Single gene analysis of *Pmepa1*, a well-known, ubiquitously expressed target of TGFβ, revealed profound downregulation in samples from arm 2, contrary to arm 3, supporting the notion of blunted TGFβ-signaling with NIS793 (Fig. 4A). Furthermore, examination of the previously identified tumor-specific NIS793 signature (11) also indicated decreased TGFβ activity in samples from patients treated with NIS793 (arm 2), contrary to what was observed in patients from arm 3 (Fig. 4B). Importantly, productive target engagement and modulation with NIS793 did not result in compensatory mechanisms leading to *de novo* expression of any TGFβ ligands, as assessed by analysis of mRNA expression for TGFβ isoforms in paired biopsies (Fig. 4C). These data demonstrate that NIS793 was effective in suppressing TGFβ signaling in tumor biopsies, supporting target engagement and positive PD in this trial.

### Cancer cell–specific changes induced by NIS793

**Figure 5.**
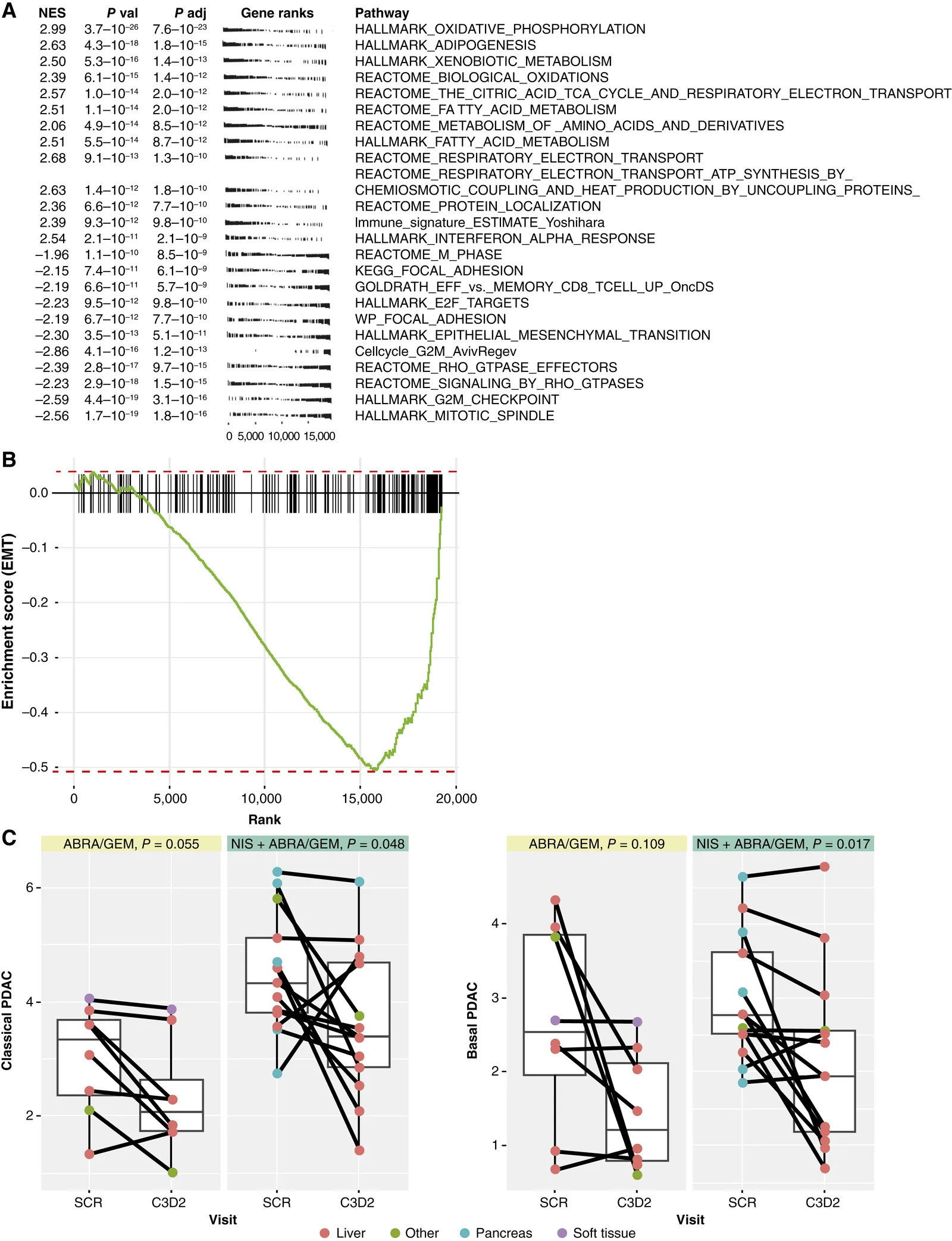
Cancer cell–intrinsic changes with NIS793. **A,** Pathways significantly affected by NIS793 treatment using GSEA from RNA-seq data of paired tissue biopsies. **B,** Analysis of EMT signatures by RNA-seq in paired biopsies. **C,** Analysis of signatures correlated with classical or basal PDAC subtype by RNA-seq in paired biopsies. *n* = 8 paired biopsies for ABRA/GEM and 13 paired biopsies for NIS793 + ABRA/GEM. NES, normalized enrichment score.

As the role of TGFβ in cancer is quite complex, due to its action on both epithelial/cancer cells as well as on the TME, we sought to explore the effects of NIS793 in paired biopsies using signatures and gene analyses to capture specific cellular compartments and biological processes. In line with the pleiotropic roles of TGFβ in cancer, GSEA of differentially expressed probes from RNA-seq data between pre- and post-treatment paired biopsies from NIS + ABRA/GEM–treated patients revealed several pathways significantly affected by NIS793 treatment, both related to cancer cell intrinsic behaviors as well as to tissue remodeling/TME dynamics (Fig. 5A).

One pathway that was found to be significantly downregulated by NIS793 related to epithelial–mesenchymal transition (EMT), a process known to be initiated by TGFβ signaling in cancer cells that furthers their metastatic dissemination potential (Fig. 5A and B). This reduction of the EMT program from NIS793 is consistent with the predicted role for TGFβ in cancer cells at later stages of disease and highlights a potential protective role for NIS793 that contrasts with the lack of benefits observed in this trial.

As these data pointed to a direct effect of NIS793 in cancer cells, they prompted an investigation of further cancer cell intrinsic features that may relate to differential tumor progression patterns. In particular, regarding PDAC, two subtypes, basal and classical, have been described and associated with different prognoses and responses to therapeutic intervention. The classical subtype has been shown to be characterized by well-differentiated tumor cells and the expression of genes characteristic of epithelial traits, and tumors with these phenotypes tend to exhibit a more organized architecture and respond better to certain therapeutic regimens, showcasing a potentially more favorable prognosis compared with the basal-like subtype. Conversely, the basal-like subtype is defined by poorly differentiated tumor cells, more aggressive behavior, and the expression of genes linked to mesenchymal traits and stemness. This subtype is linked to worse clinical outcomes and greater resistance to conventional therapies. We therefore investigated whether NIS793 may have caused a treatment-related switch between molecular subtypes, which may influence the overall reported outcome. Analysis of basal- and classical-specific RNA-seq signatures in the paired biopsies from patients treated with NIS793 + ABRA/GEM (Fig. 5C), however, showed no evidence of NIS793-driven phenotype skewing, supporting a lack of connection with the observed response. Instead, a general decrease in both basal and classical signatures was depicted in both arm 2 (NIS793 + ABRA/GEM) and arm 3 of the study (ABRA/GEM), suggesting a potential induction of a less diverse cancer cell phenotypic milieu, likely contributed to by the chemotherapy backbone. Notably, the induction of a less diverse cancer cell phenotype was previously observed also in the context of 5-FU–based chemotherapy (29), suggesting a potential common selection of more phenotypically homogeneous clones in PDAC after chemotherapy. Overall, these changes highlight a shift in cancer cell phenotypes from treatment (chemotherapy with and without NIS793) but suggest that other mechanisms are in place that limit the efficacy of NIS793 in this patient population.

### NIS793 treatment induces stromal-related changes that are consistent with Proof of Mechanism (POM)

**Figure 6.**
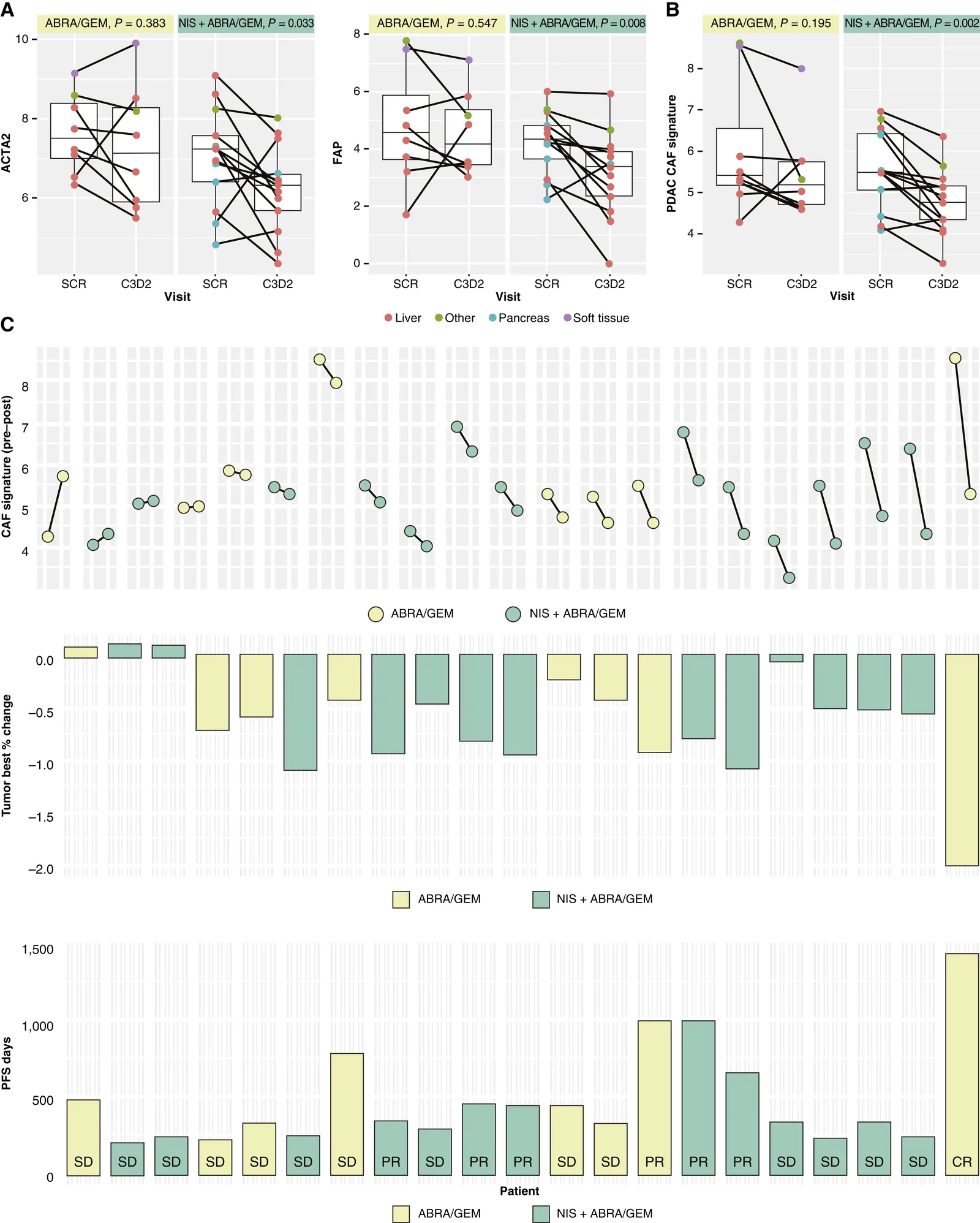
Stromal remodeling following NIS793 treatment. **A,** Downregulation of *Acta2* and *Fap* gene expression in paired biopsies from NIS793 + ABRA/GEM–treated patients (RNA-seq). **B,** Downregulation of the PDAC cancer-associated fibroblast (CAF)–specific signature in paired biopsies from NIS793 + ABRA/GEM–treated patients (RNA-seq). **C,** Correlation analysis of CAF signature change (magnitude) and response for assessable patients. *n* = 8 paired biopsies for ABRA/GEM and 13 paired biopsies for NIS793 + ABRA/GEM.

The overarching therapeutic hypothesis for NIS793 is based on the antifibrotic properties of the drug, as demonstrated by the extensive preclinical work that preceded the clinical evaluation of the drug (12). In mice, NIS793 was shown to halt the acquisition of myofibroblast traits by cancer-associated fibroblasts, limiting collagen and ECM deposition and thereby reducing tumor desmoplasia. These changes were associated with increased therapeutic benefit from either checkpoint blockade or chemotherapy treatment, also in line with the emerging view of stromal elements and intratumoral fibrosis (30). To look for stromal remodeling following NIS793 in this trial, we first analyzed expression changes of two well-characterized bona fide markers for cancer-associated fibroblasts in paired tissue biopsies. The first marker, *Acta2*, encodes alpha smooth muscle actin, an intermediate type filament protein that is uniquely expressed by contractile cells such as myofibroblasts and perivascular mesenchymal cells. The second, *Fap*, is the gene for fibroblast-activating protein, a hallmark of tumor fibroblasts that has gained considerable momentum as a promising therapeutic target for stroma in cancer. A statistically significant (*P* = 0.048 for *Acta2*; *P* = 0.013 for *Fap*) decrease for both markers in the post-treatment biopsies was readily observed in samples from arm 2, with no changes observed in arm 3 (Fig. 6A). Furthermore, we analyzed the modulation of a specific PDAC-fibroblast signature that we recently derived from single-cell RNA-seq analysis of PDAC resection (29) and similarly observed a significant decrease in the signature in arm 2 (but not in arm 3) paired biopsies (Fig. 6B). These results are of particular importance because they clearly demonstrate POM for NIS793, while underscoring the validity of the newly devised PDAC-fibroblast signature in the context of clinical data interpretation.

Notably, although a decrease in fibroblast activity and ECM production is generally correlated with a more permissive TME and better therapeutic responses, two publications have raised the controversial hypothesis that a reduction in stromal elements may instead lead to exacerbation of disease in mice (31, 32), prompting us to look at the potential correlation between the magnitude of fibroblast-related changes and patient outcome. As shown in Fig. 6C, however, no association between the magnitude of fibroblast changes and outcome could be ascertained, supporting the overall accepted notion that targeting tumor stromal cells and their bioproducts should be, if anything, positively correlated with outcomes in patients with cancer. The lack thereof in this trial likely reflects a broader effect of TGFβ blockade in other compartments beyond stroma potentially diminishing the benefits of reduced stromal activation.

### Key findings from proteomics also support PD and POM for NIS793

Proteomic profiling on longitudinal plasma samples collected from 57 subjects was performed using the SomaScan Assay version 5 (SomaLogic, Inc.), a high-throughput, aptamer-based platform designed to quantify the protein abundance of 10,070 unique human proteins in biological samples. Consistent with the transcriptomic analyses, patients treated with NIS793 showed a treatment-related change in the abundance of SOMAmers involved in TGFβ signaling (Supplementary Fig. S2A), with decreased levels observed at both cycle 3 time points compared with baseline. Notably, of the SOMAmers included in the TGFβ signature, two seemed to be most significantly affected by NIS793 treatment, namely fap_5029_3 and col1a1_1140_56, representing SOMAmers for the stromal proteins FAP and COL1A1, respectively (Supplementary Fig. S2B and S2C). Further investigation of stromal-related signatures revealed longitudinal changes in SOMAmers from different collagen species in patients who received NIS793 (Supplementary Fig. S2D), including a significant reduction in collagen type X (COL10A1) SOMAmer, col10a1_15653_9 (Supplementary Fig. S2E). Altogether, these data show some parallelism between RNA-seq and SomaScan analysis, strengthening the notion that NIS793 in patients led to effective TGFβ pathway modulation and alterations of stromal-related biological processes.

### NIS793 treatment may result in the promotion of neutrophil-related pathways

**Figure 7.**
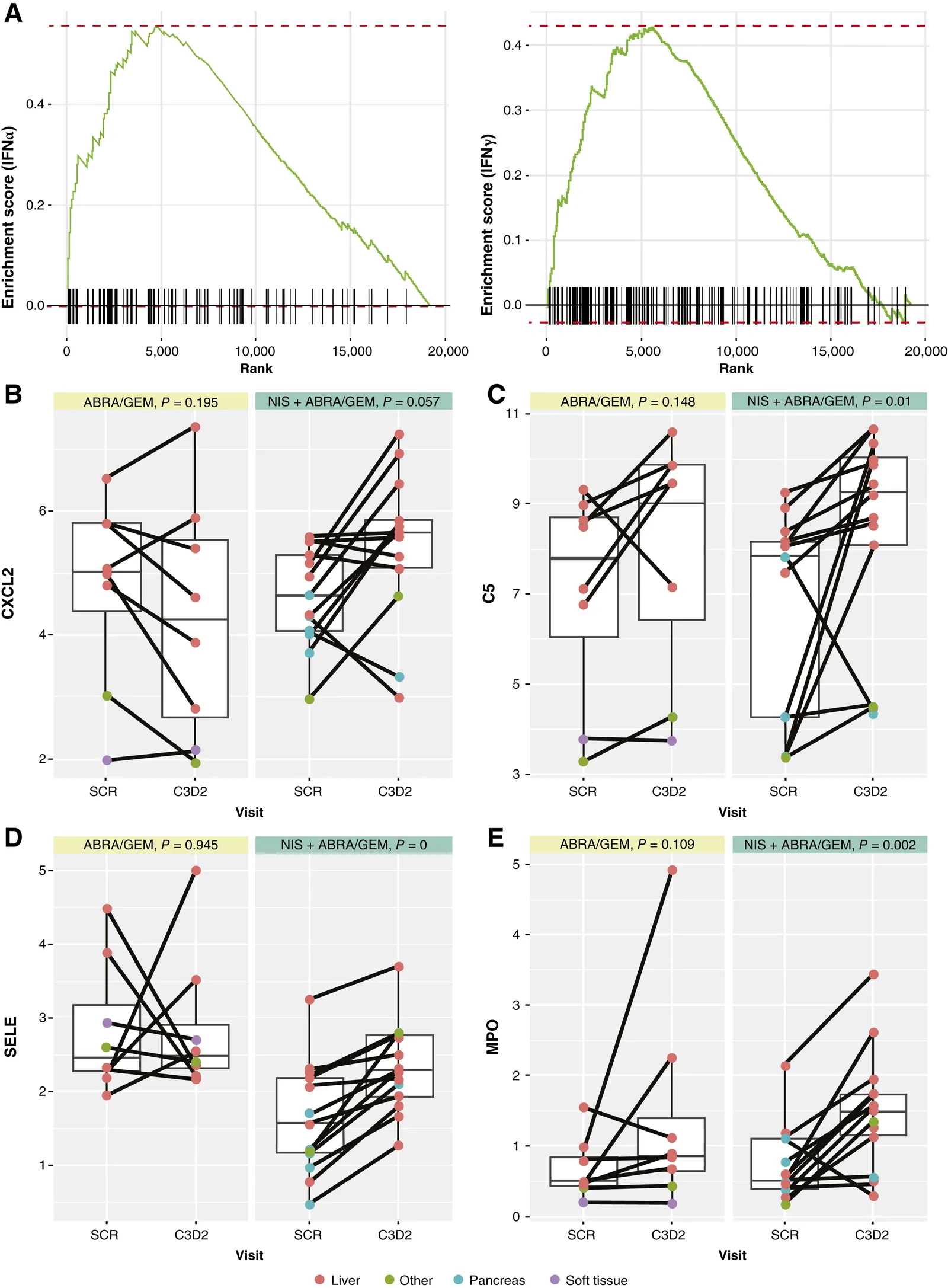
Upregulation of innate immune markers following NIS793 treatment. **A,** IFNα and IFNγ signature scores in paired biopsies from arm 2 compared with baseline (RNA-seq). **B–E,** Changes in the expression of the depicted genes as measured by RNA-seq in paired biopsies. *n* = 8 paired biopsies for ABRA/GEM and 13 paired biopsies for NIS793 + ABRA/GEM. *Mpo*, myeloperoxidase.

Together with the opportunity to modulate stromal behavior in desmoplastic tumors, the appeal around TGFβ targeting for cancer also stems from its immunosuppressive role in adaptive immune cells, particularly T cells, and the potential of unleashing antitumor cytotoxic responses. In line with this notion, NIS793 was found to drive the expression of genes indicative of IFN responses and immune activity (Figs. 5A and 7A), consistent with data previously generated both in preclinical models (12) and in the FIH trial with NIS793 (11).

However, TGFβ’s immunomodulatory range of action spans beyond T cells, and a plethora of studies have also demonstrated a role for TGFβ in the regulation of innate immune pathways with context-dependent outcomes. One aspect that has been postulated by these studies is the function of TGFβ in limiting neutrophil recruitment and activity, a role that is critical to promote the resolution of inflammation and restore tissue homeostasis. Based on these facts, we explored whether NIS793 inadvertently led to the exacerbation of neutrophil-related pathways, a phenomenon that, in the context of a growing cancer, may, in certain cases, instigate the development of tumor-promoting inflammation. Indeed, RNA-seq data from paired biopsies suggested that several genes implicated in neutrophil function were increased with treatment in arm 2 compared with arm 3. *Cxcl2* and *C5*, for example, both molecules associated with neutrophil chemotaxis to inflamed tissues, were significantly increased after NIS793 treatment (Fig. 7B and C), as was *Sele* (Fig. 7D), the gene encoding E-selectin, an adhesion molecule mediating the adhesion and rolling of leukocytes on endothelial cells during extravasation. Similarly, the gene for myeloperoxidase, an enzyme contained in neutrophil secondary granules, was also enriched in samples from patients who received NIS793 (Fig. 7E). What these data suggest is that NIS793 treatment may potentiate local innate immune activation, in particular neutrophils, and are consistent with previous reports showing suppression of *Cxcl2* expression by TGFβ leading to limited neutrophil recruitment (33) and other studies demonstrating TGFβ-mediated downregulation of E-selectin expression on endothelial cells limiting neutrophil extravasation (34, 35). Notably, increased neutrophil recruitment and activation were also observed in preclinical studies using NIS793 (Supplementary Fig. S3; ref. 12). As these pathways can contribute to a persistent inflammatory environment that could ultimately fuel tumor growth, it is possible that the effects of NIS793, as evidenced by this trial (and the phase III pivotal trial), may result from the induction of tumor-promoting inflammation. This potential mechanism, however, remains to be further validated.

## Discussion

Following the completion of the phase Ib trial of NIS793 (11), a phase II study in mPDAC was initiated. This decision was based on the mechanistic hypothesis, supported by preclinical validation (12), that TGFβ blockade with NIS793 may offer significant combinatorial activity in highly desmoplastic tumors by remodeling stromal elements and halting the development of intratumoral fibrosis. However, as reported here, a trend toward decreased OS was observed in patients receiving NIS793 alongside ABRA/GEM. A review of available biomarker data from this trial, combined with an examination of preclinical parameters relevant to the mechanism of action of NIS793, revealed a pharmacologic profile consistent with NIS793 as a drug asset. The collective findings supported target engagement and POM but also revealed increased expression of markers associated with innate immune activity. Although a direct correlation between the clinical outcomes and the underlying molecular mechanisms cannot be conclusively drawn from these analyses, this study contributes to the growing understanding of the complexity involved in targeting TGFβ in cancer, underscoring the challenges associated with the clinical use of untargeted TGFβ blockers.

The cytostatic effects of TGFβ on cancer cells during the early stages of tumor development are well established (36). However, sustained TGFβ signaling within the TME has been associated with cellular and tissue alterations that promote cancer cell growth, metastasis, and tumor-associated immune suppression (37, 38). This concept has been extensively validated in preclinical models and has supported the exploration of therapeutic strategies aimed at reducing TGFβ activity in patients with established disease (39). Studies from multiple groups have demonstrated the potential of TGFβ inhibition to enhance the efficacy of other treatments, including checkpoint inhibitors, radiotherapy, and chemotherapy, underscoring the promise of TGFβ-blocking agents as valuable components of combination therapies in oncology (40, 41). However, clinical data have shown variable outcomes, highlighting the challenges of targeting TGFβ in clinical settings. Galunisertib, one of the earliest and most extensively studied small-molecule inhibitors of TGFβRI kinase activity, yielded mixed results in clinical trials (42) and was discontinued by Eli Lilly in 2020 although some trials remain ongoing. Fresolimumab, a mAb targeting all isoforms of TGFβ, showed promise in early-phase clinical trials for melanoma, renal cell carcinoma, and non–small cell lung cancer, but it encountered significant hurdles in later-stage trials, leading to its discontinuation in oncology (43). More recently, bintrafusp alfa, a bifunctional fusion protein targeting both TGFβ and PD-L1, was also discontinued due to modest activity that seemed limited to specific cancer types and patient populations (44). In this context, the lack of clinical benefit observed with NIS793 in the present study aligns with findings from other TGFβ-targeting agents, further illustrating the difficulty of translating preclinical success into clinical benefit.

One aspect brought to light by this study was that the addition of NIS793 to ABRA/GEM for mPDAC might have a potential negative impact on OS. Although this effect was not statistically significant in the current study, the possibility of a detrimental outcome was reinforced by clinical findings from a parallel phase III trial of NIS793, in which a marked reduction in both PFS and OS was reported in the combination arm (ClinicalTrials.gov ID NCT04935359, manuscript under consideration). Additional reports of a diminished benefit/risk profile from TGFβ blockade with other agents have also been documented. These include an increased incidence of early progression or death in certain patient populations treated with bintrafusp alfa although such findings were limited to individual cases or small cohorts (43). Collectively, these data underscore the complexity of targeting TGFβ and raise concerns about the feasibility and potential liabilities of such approaches. Notably, the use of NIS793 was not associated with significant TRAEs or DLTs, and preclinical toxicology data indicated an acceptable safety profile at clinically relevant doses, indicating a potential role for NIS793 in tumor progression as a possible contributor to the observed clinical outcomes.

The failure of NIS793 to demonstrate clinical efficacy did not seem to be due to unusual antibody properties or imbalances in patient characteristics in the herein described trial. Nor did it seem to be linked to tumor cell effects that might promote more aggressive behaviors. Instead, the data showed a correlation with reduced EMT phenotype in patients receiving NIS793, consistent with the known role of TGFβ in cancer cells. From the perspective of the TME, NIS793 was effective in modulating stromal cell dynamics, leading to a significant downregulation of markers associated with cancer-associated fibroblasts. These findings are important because they reflect preclinical data generated with NIS793 and support the mechanistic hypothesis that TGFβ blockade can effectively reduce the desmoplastic reaction in tumors (11, 13). In preclinical studies, these stromal effects were associated with improved responses to PD-1 blockade or chemotherapy (12, 13), raising the question of why, in the clinical trial reported here, successful stroma modulation did not translate into better outcomes. Importantly, the extent of fibroblast modulation did not directly correlate with poorer outcomes, suggesting that although stroma remodeling did not translate into better clinical benefit, it did not worsen it either. Indeed, previous trials using agents with antifibrotic properties in PDAC have shown safety despite modest antitumor activity. For example, PEGPH20, a pegylated recombinant human hyaluronidase, was shown to degrade intratumoral hyaluronic acid and remodel the tumor stroma, resulting in increased PFS when combined with gemcitabine and nab-paclitaxel in its phase II trial (45) but failed to meet endpoints in the phase III trial (46). Taken together, these data raise important questions on the heterogeneity of stroma in PDAC and implications for targeting and suggest that although NIS793 may be effective in reducing stromal activation in tumors, other factors within the TME, likely not well replicated in animal models, may contribute to the clinical outcomes observed (47).

Increased expression of genes linked to neutrophil recruitment and activity in paired biopsies, alongside enhanced neutrophil infiltration and activation in NIS793-treated mice, indicates a role for TGFβ in innate immune activation. As TGFβ is known to suppress neutrophil recruitment and activation during inflammation to promote the resolution of the inflammatory response and restore tissue balance, TGFβ blockade may indeed result in unrestrained neutrophil-related immunologic responses. It is intriguing to speculate that this neutrophil influx may disrupt immune equilibrium within the TME, triggering immunosuppressive, tumor-supporting neutrophil behaviors, potentially amplified by concurrent complement activation. In this context, innate immune priming by NIS793 may lead to a TME characterized by chronic inflammation, which can fuel cancer cell growth, angiogenesis, and metastasis. This hypothesis could help explain the decreased OS observed in arm 2. It may also account for the reported efficacy in the 4T1 tumor model despite neutrophil influx, as tumor-promoting inflammation is a gradual accumulation of traits within the TME that only manifest over time. This may not be accurately captured in short-lived preclinical models. Further studies are needed to formally establish causality for these observations.

In conclusion, this study presents the clinical and biomarker findings from the NIS793 phase II randomized trial in mPDAC, highlighting the complexity of TGFβ targeting, even with a molecule with well-controlled pharmacologic properties like NIS793. Together with NIS793 phase III data, these findings warrant caution about TGFβ blockade in cancer and suggest that benefits relative to risks should be carefully weighed in future clinical explorations of TGFβ blockade as a potential anticancer therapeutic approach.

## Supplementary Material

Supplementary Data1 Supplementary Tables and Figures

Supplementary Data2 Study representativeness table

## Data Availability

All relevant data are included in the article and its Supplemental Information files. Patient-level data cannot be shared because of privacy considerations. The genomic data generated in this study are not publicly available because they contain potentially identifiable patient-level information and are subject to clinical trial consent and privacy restrictions. Data may be made available from the corresponding author upon reasonable request, subject to appropriate institutional approval and data use agreements.
