# Supplementary material for: Biomarker Analysis from Patients with Metastatic PDAC Treated with TGFβ Antibody NIS793 plus Abraxane + Gemcitabine versus Abraxane + Gemcitabine Alone in a Phase II, Open-Label, Randomized Study
Source: Clin Cancer Res. 2026 Jun 25;32(18):4001–15. doi: 10.1158/1078-0432.CCR-26-0805 (PMC13575558; doi:10.1158/1078-0432.CCR-26-0805)
Supplement: Supplementary file 1 [file ccr-26-0805_supplementary_data1_suppds1.docx]

# **Supplementary figures and tables**

## Supplementary Figure 1. Study design. After Safety Run-in to assess the safety and tolerability of NIS793 in combination with spartalizumab and gemcitabine/nab-paclitaxel, participants were randomized in a 1:1:1 ratio to one of the three treatment arms: arm 1, NIS793 with spartalizumab and gemcitabine/nab-paclitaxel; arm 2, NIS793 with gemcitabine/nab-paclitaxel; arm 3, gemcitabine/nab-paclitaxel.

## Supplementary Figure 2. SOMAlogic analysis of TGFβ marker protein levels in mPDAC (SomaScan). Longitudinal changes for SOMAmers from A. TGFβ signature, B. FAP, C. COL1A1, D. collagen formation module and E. COL10A1 across the two arms. Statistical significance was determined by two-sided, paired t-test for each timepoint compared to Cycle 1 Day 1 (C1D1).

A. **B.**

**C. D. E.**

**Supplementary Figure 3: Neutrophil recruitment in 4T1 tumor-bearing mice treated with NIS793 *in vivo*.** 4T1 tumor bearing mice were dosed with NIS793 as described in Grauel et al., Nat. Commun., 2020, and tumors were excised at the end of experiment and analyzed by flow cytometry. Plot depicts quantification of neutrophils from dissociated tumors. Each dot represents a mouse. Mean is depicted. n = 10 mice per group. P value was calculated using unpaired, two-tailed t-test with Welch’s correction.

## Supplementary Table 1. Patient demographics.

*Safety run in part*

| **Characteristic** | **NIS793 with spartalizumab and gemcitabine/nab-paclitaxel**  **N=11** |
| --- | --- |
| **Age group (18-<65 years) - n (%)** | 5 (45.5) |
| **Age (years)** |  |
| **Mean (SD)** | 63.5 (8.77) |
| **Median** | 65.0 (41 – 73) |
| **Sex (Male) – n(%)** | 6 (54.5) |
| **Race – n (%)** |  |
| **White** | 9 (81.8) |
| **Asian (Chinese)** | 2 (18.2) |
| **Ethnicity – n (%)** |  |
| **Hispanic Or Latino** | 1 (9.1) |
| **Not Hispanic or Latino** | 10 (90.9) |
| **Weight (kg)** |  |
| **Mean (SD)** | 75.6 (22.19) |
| **Median** | 71.4 (55.0 -120.0) |
| **Height (cm)** |  |
| **Mean (SD)** | 171.2 (10.07) |
| **Median** | 174.0 (149.0 -184.0) |
| **Body Mass Index(kg/m2)** |  |
| **Mean (SD)** | 25.7 (6.72) |
| **Median** | 23.8 (18 – 42) |
| **ECOG*-n (%)** |  |
| **0** | 7 (63.6) |
| **1** | 4 (36.4) |

| *Randomized part* | | | | |
| --- | --- | --- | --- | --- |
| Characteristic | NIS793 with spartalizumab and gemcitabine/nab-paclitaxel N=50 | NIS793 with gemcitabine/nab-paclitaxel N=51 | Gemcitabine/nab-paclitaxel N=52 | |
| **Age group (18- <65 years) – n (%)** | 20 (40.0) | 23 (45.1) | | 23 (44.2) |
| **Age (years)** |  |  | |  |
| **Mean (SD)** | 64.3 (10.91) | 64.2 (9.90) | | 64.8 (8.25) |
| **Median** | 67.0 (34 – 82) | 65.0 (27 – 79) | | 66.0 (34 – 82) |
| **Sex (Male) – n (%)** | 29 (58.0) | 30 (58.8) | | 32 (61.5) |
| **Race – n (%)** |  |  | |  |
| **White** | 39 (78.0) | 35 (68.6) | | 33 (63.5) |
| **Black or African American** | 1 (2.0) | 1 (2.0) | | 3 (5.8) |
| **Asian** | 9 (18.0) | 15 (29.41) | | 15 (28.85) |
| **Chinese** | 8 (16.0) | 13 (25.5) | | 11 (21.2) |
| **Indian** | 1 (2.0) | 1 (2.0) | | 2 (3.8) |
| **Missing** | 0 | 1 (2.0) | | 2 (3.8) |
| **Unknown** | 1 (2.0) | 0 | | 1 (1.9) |
| **Ethnicity – n (%)** |  |  | |  |
| **Hispanic or Latino** | 3 (6.0) | 1 (2.0) | | 2 (3.8) |
| **Not Hispanic or Latino** | 40 (80.0) | 47 (92.2) | | 47 (90.4) |
| **Not reported** | 4 (8.0) | 1 (2.0) | | 2 (3.8) |
| **Unknown** | 3 (6.0) | 2 (3.9) | | 1 (1.9) |
| **Weight (kg)** |  |  | |  |
| **Mean (SD)** | 72.9 (14.77) | 70.3 (17.70) | | 70.1 (14.80) |
| **Median** | 72.8 (49.0 -113.0) | 67.0 (39.0 -130.0) | | 70.3 (44.0 -101.0) |
| **Height (cm)** |  |  | |  |
| **Mean (SD)** | 168.8 (8.88) | 167.2 (10.86) | | 169.0 (9.07) |
| **Median** | 168.0 (150.0 -192.0) | 168.0 (147.0 -202.0) | | 170.0 (152.0 -187.0) |
| **Body Mass Index(kg/m^2^)** |  |  | |  |
| **Mean (SD)** | 25.5 (4.38) | 25.0 (4.81) | | 24.4 (4.16) |
| **Median** | 24.7 (17 – 37) | 23.9 (17 – 40) | | 23.8 (15 – 35) |
| **ECOG*-n (%)** |  |  | |  |
| **0** | 24 (48.0) | 28 (54.9) | | 22 (42.3) |
| **1** | 26 (52.0) | 23 (45.1) | | 30 (57.7) |

## Supplementary Table 2. Baseline disease characteristics.

*Safety run in part*

| **Characteristic** | **NIS793 with spartalizumab and gemcitabine/nab-paclitaxel N=11** |
| --- | --- |
| **Primary site of cancer -n (%)** |  |
| **Pancreas** | 10 (90.9) |
| **Pancreatic Duct** | 1 (9.1) |
| **Histology/cytology -n (%)** |  |
| **Adenocarcinoma** | 11 (100) |
| **Histological grade -n (%)** |  |
| **Well Differentiated** | 2 (18.2) |
| **Moderately Differentiated** | 2 (18.2) |
| **Poorly Differentiated** | 4 (36.4) |
| **Unknown** | 3 (27.3) |
| **Stage IV at initial diagnosis -n (%)** | 11 (100) |
| **Time since initial diagnosis of primary site (months)** |  |
| **Mean (SD)** | 1.1 (0.46) |
| **Median** | 1.0 (0 – 2) |
| **Stage IV at time of study entry -n (%)** | 11 (100) |
| **Extent of disease at the time of study entry-n (%)** |  |
| **Adrenal** | 1 (9.1) |
| **Ascites (Malignant)** | 4 (36.4) |
| **Bone** | 1 (9.1) |
| **Liver** | 10 (90.9) |
| **Pancreas** | 1 (9.1) |
| **Retroperitoneal Lymph Nodes** | 2 (18.2) |
| **Spleen** | 1 (9.1) |
| **Other** | 5 (45.5) |

| *Randomized trial part* |  | | |
| --- | --- | --- | --- |
| **Categories/Statistics** | **NIS793 with spartalizumab and gemcitabine/ nab-paclitaxel N=50** | **NIS793 with gemcitabine/nab-paclitaxel N=51** | **Gemcitabine/nab-paclitaxel N=52** |
| **Primary site of cancer-n (%)** |  |  |  |
| **Pancreatic Head** | 1 (2.0) | 1 (2.0) | 0 |
| **Pancreas** | 44 (88.0) | 40 (78.4) | 46 (88.5) |
| **Pancreatic Duct** | 5 (10.0) | 10 (19.6) | 6 (11.5) |
| **Histology/cytology-n (%)** |  |  |  |
| **Adenocarcinoma** | 49 (98.0) | 51 (100) | 52 (100) |
| **Missing** | 1 (2.0) | 0 | 0 |
| **Histological grade-n (%)** |  |  |  |
| **Well Differentiated** | 10 (20.0) | 8 (15.7) | 8 (15.4) |
| **Moderately Differentiated** | 17 (34.0) | 19 (37.3) | 15 (28.8) |
| **Poorly Differentiated** | 7 (14.0) | 5 (9.8) | 7 (13.5) |
| **Unknown** | 16 (32.0) | 19 (37.3) | 22 (42.3) |
| **Stage at initial diagnosis-n (%)** |  |  |  |
| **IA** | 0 | 1 (2.0) | 0 |
| **IIA** | 1 (2.0) | 0 | 0 |
| **III** | 2 (4.0) | 1 (2.0) | 1 (1.9) |
| **IV** | 47 (94.0) | 49 (96.1) | 51 (98.1) |
| **Time since initial diagnosis of primary site (months)** |  |  |  |
| **Mean (SD)** | 2.0 (3.69) | 1.2 (0.60) | 1.3 (0.84) |
| **Median** | 1.1 (0 – 24) | 1.1 (0 – 3) | 1.1 (0 – 6) |
| **Stage IV at time of study entry-n (%)** | 50 (100) | 51 (100) | 52 (100) |
| **Extent of disease at time of study entry-n (%)** |  |  |  |
| **Adrenal** | 2 (4.0) | 0 | 2 (3.8) |
| **Anterior Mediastinal Lymph Nodes** | 1 (2.0) | 1 (2.0) | 2 (3.8) |
| **Ascites (Malignant)** | 4 (8.0) | 1 (2.0) | 2 (3.8) |
| **Axillary Lymph Nodes** | 0 | 0 | 1 (1.9) |
| **Bone** | 1 (2.0) | 4 (7.8) | 3 (5.8) |
| **Cervical Lymph Nodes** | 1 (2.0) | 0 | 1 (1.9) |
| **Liver** | 43 (86.0) | 42 (82.4) | 45 (86.5) |
| **Lung** | 19 (38.0) | 19 (37.3) | 10 (19.2) |
| **Pancreas** | 3 (6.0) | 1 (2.0) | 6 (11.5) |
| **Para-Aortic Lymph Nodes** | 4 (8.0) | 6 (11.8) | 4 (7.7) |
| **Pleural Effusion (Malignant)** | 0 | 0 | 1 (1.9) |
| **Pulmonary Lymph Nodes** | 0 | 1 (2.0) | 2 (3.8) |
| **Retroperitoneal Lymph Nodes** | 5 (10.0) | 6 (11.8) | 3 (5.8) |
| **Spleen** | 3 (6.0) | 1 (2.0) | 3 (5.8) |
| **Other** | 24 (48.0) | 26 (51.0) | 17 (32.7) |

## Supplementary Table 3. Patient disposition. A total of 164 participants were enrolled in the study. Of these 164 participants, 11 were in the Safety run-in part and 153 in the Randomized part. Reasons for discontinuation are reported in the tables below.

*Safety run in part*

| **Disposition/Reason** | **NIS793 with spartalizumab and gemcitabine/nab-paclitaxel N=11  (n%)** |
| --- | --- |
| **Participants treated** |  |
| **Treated** | 11 (100) |
| **Treatment discontinued** | 11 (100) |
| **Primary reason for end of treatment** |  |
| **Adverse event** | 4 (36.4) |
| **Physician Decision** | 1 (9.1) |
| **Progressive Disease** | 6 (54.5) |
| **Post-treatment follow-up for participants who discontinued treatment** |  |
| **Entered post-treatment follow-up discontinued** | 3 (27.3) |
| **Reason for discontinuation** |  |
| **Death** | 2 (18.2) |
| **Progressive Disease** | 1 (9.1) |

| *Randomized part* | | | | | | |  |
| --- | --- | --- | --- | --- | --- | --- | --- |
| **Disposition/Reason** | **NIS793 with spartalizumab and gemcitabine/nab-paclitaxel N=50 (n%)** | | **NIS793 with gemcitabine/nab-paclitaxel N=51 (n%)** | | **Gemcitabine/nab-paclitaxel N=52 (n%)** | |  |
| **Participants treated** |  | |  | |  | |  |
| **Treated** | 46 (92.0) | | 49 (96.1) | | 45 (86.5) | |  |
| **Not treated** | 4 (8.0) | | 2 (3.9) | | 7 (13.5) | |  |
| **Reason for not being treated** |  | |  | |  | |  |
| **Adverse Event** | 2 (4.0) | | 1 (2.0) | | 1 (1.9) | |  |
| **Physician Decision** | 2 (4.0) | | 0 | | 0 | |  |
| **Participant Decision** | 0 | | 1 (2.0) | | 6 (11.5) | |  |
| **Treatment discontinued** | 46 (92.0) | | 49 (96.1) | | 45 (86.5) | |  |
| **Primary reason for end of treatment** | | | | | | | |
| **Adverse Event** | | 11 (22.0) | | 5 (9.8) | | 5 (9.6) |  |
| **Death** | | 3 (6.0) | | 4 (7.8) | | 2 (3.8) |  |
| **Physician Decision** | | 2 (4.0) | | 2 (3.9) | | 9 (17.3) |  |
| **Progressive Disease** | | 24 (48.0) | | 31 (60.8) | | 23 (44.2) |  |
| **Study terminated by sponsor** | | 1 (2.0) | | 0 | | 0 |  |
| **Participant Decision** | | 5 (10.0) | | 7 (13.7) | | 6 (11.5) |  |
| **Post-treatment follow-up for participants who discontinued treatment** | | | | | | | |
| **Entered post-treatment follow-up, discontinued** | | 9 (18.0) | | 4 (7.8) | | 13 (25.0) |  |
| **Reason for discontinuation** | |  | |  | |  |  |
| **Death** | | 1 (2.0) | | 0 | | 4 (7.7) |  |
| **Progressive Disease** | | 7 (14.0) | | 3 (5.9) | | 9 (17.3) |  |
| **Study terminated by sponsor** | | 1 (2.0) | | 1 (2.0) | | 0 |  |

## Supplementary Table 4. Dose-limiting toxicities. Numbers (n) represent counts of participants. AEs occurring during treatment or within 30 days of the last study drug are summarized. MedDRA version 27.0

*Safety run in part*

|  | NIS793 with  spartalizumab and  gemcitabine/nab-  paclitaxel |
| --- | --- |
| Primary System Organ Class | N=6 |
| Preferred term | n (%) |
| Number of participants with at least one event | 1 (16.7) |
| Gastrointestinal disorders | 1 (16.7) |
| Colitis | 1 (16.7) |

## Supplementary Table 5. Overview of adverse events. Numbers (n) represent counts of participants. A participant with multiple severity grades for an AE is only counted under the maximum grade. MedDRA version 27.0, CTCAE version 5.0.

*Safety run in part*

| Category (Safety run-in) | NIS793 with spartalizumab and gemcitabine/nab-paclitaxel N=11 n (%) |
| --- | --- |
| Adverse events | 11 (100) |
| Treatment-related | 11 (100) |
| AEs with grade ≥3 | 11 (100) |
| Treatment-related | 8 (72.7) |
| SAEs | 8 (72.7) |
| Treatment-related | 3 (27.3) |
| Fatal SAEs | 1 (9.1) |
| Treatment-related | 0 |
| AEs leading to study drug discontinuation | 4 (36.4) |
| Treatment-related | 2 (18.2) |
| AEs leading to dose adjustment/interruption | 9 (81.8) |
| AEs requiring additional therapy | 11 (100) |

*Randomized part*

| Category | NIS793 with spartalizumab and gemcitabine/nab-paclitaxel N=46 n (%) | NIS793 with gemcitabine/nab-paclitaxel N=49 n (%) | Gemcitabine/nab-paclitaxel N=45 n (%) | All participants  N=140  n (%) |
| --- | --- | --- | --- | --- |
| Adverse events | 45 (97.8) | 49 (100) | 43 (95.6) | 137 (97.9) |
| Treatment-related | 45 (97.8) | 45 (91.8) | 38 (84.4) | 128 (91.4) |
| AEs with grade ≥3 | 42 (91.3) | 42 (85.7) | 35 (77.8) | 119 (85.0) |
| Treatment-related | 34 (73.9) | 37 (75.5) | 24 (53.3) | 95 (67.9) |
| SAEs | 32 (69.6) | 26 (53.1) | 26 (57.8) | 84 (60.0) |
| Treatment-related | 19 (41.3) | 12 (24.5) | 12 (26.7) | 43 (30.7) |
| Fatal SAEs | 3 (6.5) | 1 (2.0) | 4 (8.9) | 8 (5.7) |
| Treatment-related | 1 (2.2) | 0 | 0 | 1 (0.7) |
| AEs leading to study drug discontinuation | 7 (15.2) | 4 (8.2) | 5 (11.1) | 16 (11.4) |
| Treatment-related | 5 (10.9) | 4 (8.2) | 3 (6.7) | 12 (8.6) |
| AEs leading to dose adjustment/interruption | 40 (87.0) | 37 (75.5) | 33 (73.3) | 110 (78.6) |
| AEs requiring additional therapy | 45 (97.8) | 48 (98.0) | 40 (88.9) | 133 (95.0) |

## Supplementary Table 6. Treatment related AEs. Numbers (n) represent counts of participants. AEs occurring during treatment or within 30 days of the last study drug are summarized. A participant with multiple severity grades for an AE is only counted under the maximum grade/ severity. MedDRA version 27.0, CTCAE version 5.0.

*Safety run in part*

|  | NIS793 with  spartalizumab and  gemcitabine/nab-  paclitaxel  N=11 | |
| --- | --- | --- |
|  | **All Grades** | **Grade ≥3** |
| Preferred term | **n (%)** | **n (%)** |
| Alopecia | 3 (27.3) | 0 |
| Decreased appetite | 3 (27.3) | 0 |
| Blood alkaline phosphatase increased | 2 (18.2) | 1 (9.1) |
| Epistaxis | 2 (18.2) | 0 |
| Gamma-glutamyltransferase increased | 2 (18.2) | 1 (9.1) |
| Gingival bleeding | 2 (18.2) | 0 |
| Gingival swelling | 2 (18.2) | 0 |
| Neutrophil count decreased | 2 (18.2) | 1 (9.1) |
| Paraesthesia | 2 (18.2) | 0 |
| Rash maculo-papular | 2 (18.2) | 2 (18.2) |
| Thrombocytopenia | 2 (18.2) | 0 |
| Vomiting | 2 (18.2) | 0 |
| Colitis | 1 (9.1) | 1 (9.1) |
| Diarrhoea haemorrhagic | 1 (9.1) | 1 (9.1) |
| Hepatobiliary disease | 1 (9.1) | 1 (9.1) |
| Venous thrombosis | 1 (9.1) | 1 (9.1) |
| Weight decreased | 1 (9.1) | 1 (9.1) |

*Randomized part*

|  | NIS793 with  spartalizumab and  gemcitabine/nab-  paclitaxel  N=46 | | NIS793 with  gemcitabine/nab-  paclitaxel  N=49 | | Gemcitabine/nab-  paclitaxel  N=45 | | All Participants  N=140 | |
| --- | --- | --- | --- | --- | --- | --- | --- | --- |
|  | **All Grades** | **Grade ≥3** | **All Grades** | **Grade ≥3** | **All Grades** | **Grade ≥3** | **All Grades** | **Grade ≥3** |
| Preferred term | **n (%)** | **n (%)** | **n (%)** | **n (%)** | **n (%)** | **n (%)** | **n (%)** | **n (%)** |
| Number of participants with at least one event | 45 (97.8) | 34 (73.9) | 45 (91.8) | 37 (75.5) | 38 (84.4) | 24 (53.3) | 128 (91.4) | 95 (67.9) |
| Anaemia | 25 (54.3) | 13 (28.3) | 28 (57.1) | 17 (34.7) | 16 (35.6) | 7 (15.6) | 69 (49.3) | 37 (26.4) |
| Nausea | 15 (32.6) | 1 (2.2) | 19 (38.8) | 0 | 15 (33.3) | 2 (4.4) | 49 (35.0) | 3 (2.1) |
| Diarrhoea | 19 (41.3) | 2 (4.3) | 12 (24.5) | 0 | 13 (28.9) | 0 | 44 (31.4) | 2 (1.4) |
| Fatigue | 18 (39.1) | 2 (4.3) | 15 (30.6) | 2 (4.1) | 11 (24.4) | 1 (2.2) | 44 (31.4) | 5 (3.6) |
| Asthenia | 9 (19.6) | 1 (2.2) | 14 (28.6) | 1 (2.0) | 11 (24.4) | 3 (6.7) | 34 (24.3) | 5 (3.6) |
| Pyrexia | 16 (34.8) | 1 (2.2) | 5 (10.2) | 0 | 11 (24.4) | 0 | 32 (22.9) | 1 (0.7) |
| Rash | 11 (23.9) | 1 (2.2) | 14 (28.6) | 0 | 5 (11.1) | 1 (2.2) | 30 (21.4) | 2 (1.4) |
| Alopecia | 8 (17.4) | 0 | 14 (28.6) | 2 (4.1) | 7 (15.6) | 0 | 29 (20.7) | 2 (1.4) |
| Neutropenia | 12 (26.1) | 8 (17.4) | 8 (16.3) | 5 (10.2) | 9 (20.0) | 7 (15.6) | 29 (20.7) | 20 (14.3) |
| Vomiting | 7 (15.2) | 0 | 13 (26.5) | 0 | 9 (20.0) | 1 (2.2) | 29 (20.7) | 1 (0.7) |
| Decreased appetite | 6 (13.0) | 0 | 11 (22.4) | 0 | 10 (22.2) | 0 | 27 (19.3) | 0 |
| Platelet count decreased | 8 (17.4) | 3 (6.5) | 10 (20.4) | 2 (4.1) | 8 (17.8) | 2 (4.4) | 26 (18.6) | 7 (5.0) |
| Alanine aminotransferase increased | 11 (23.9) | 2 (4.3) | 6 (12.2) | 2 (4.1) | 6 (13.3) | 2 (4.4) | 23 (16.4) | 6 (4.3) |
| Neutrophil count decreased | 6 (13.0) | 5 (10.9) | 8 (16.3) | 7 (14.3) | 9 (20.0) | 5 (11.1) | 23 (16.4) | 17 (12.1) |
| Neuropathy peripheral | 5 (10.9) | 0 | 11 (22.4) | 1 (2.0) | 5 (11.1) | 1 (2.2) | 21 (15.0) | 2 (1.4) |
| Aspartate aminotransferase increased | 10 (21.7) | 2 (4.3) | 5 (10.2) | 0 | 4 (8.9) | 1 (2.2) | 19 (13.6) | 3 (2.1) |
| Epistaxis | 7 (15.2) | 0 | 10 (20.4) | 2 (4.1) | 1 (2.2) | 0 | 18 (12.9) | 2 (1.4) |
| Pruritus | 11 (23.9) | 0 | 5 (10.2) | 0 | 1 (2.2) | 0 | 17 (12.1) | 0 |
| Oedema peripheral | 3 (6.5) | 0 | 8 (16.3) | 0 | 3 (6.7) | 0 | 14 (10.0) | 0 |

## Supplementary Table 7. AESIs. Numbers (n) represent counts of participants. A participant with multiple severity grades for an AE is only counted under the maximum grade. MedDRA version 27.0, CTCAE version 5.0. Case Retrieval Strategy version released 03-JUL-2024. *The 2 PTs Hydrocele (arm 1) and mastitis fungal (arm 2) were pulled because of the broad search criteria and they are not true embryo fetal toxicity. Please add the Listing related to this information.

*Safety run in part*

| Safety run-in | NIS793 with  spartalizumab and  gemcitabine/nab-  paclitaxel  N=11 | |
| --- | --- | --- |
|  | All Grades | Grade ≥3 |
| Safety topic | n (%) | n (%) |
| Infusion related reactions | 11 (100) | 2 (18.2) |
| Skin Toxicity | 9 (81.8) | 2 (18.2) |
| Hemorrhage | 4 (36.4) | 2 (18.2) |
| Cardiac disorder | 2 (18.2) | 0 |
| Vascular disorders | 1 (9.1) | 0 |

*Randomized part*

|  | NIS793 with  spartalizumab and  gemcitabine/nab-  paclitaxel  N=46 | | | NIS793 with  gemcitabine/nab-  paclitaxel  N=49 | | All Participants  N=95 | | |
| --- | --- | --- | --- | --- | --- | --- | --- | --- |
|  | All Grades | Grade ≥3 | All Grades | | Grade ≥3 | | All Grades | Grade ≥3 |
| Safety topic | n (%) | n (%) | n (%) | | n (%) | | n (%) | n (%) |
| Infusion related reactions | 41 (89.1) | 8 (17.4) | 45 (91.8) | | 10 (20.4) | | 86 (90.5) | 18 (18.9) |
| Skin Toxicity | 33 (71.7) | 2 (4.3) | 32 (65.3) | | 4 (8.2) | | 65 (68.4) | 6 (6.3) |
| Hemorrhage | 20 (43.5) | 5 (10.9) | 25 (51.0) | | 7 (14.3) | | 45 (47.4) | 12 (12.6) |
| Cardiac disorder | 9 (19.6) | 1 (2.2) | 14 (28.6) | | 4 (8.2) | | 23 (24.2) | 5 (5.3) |
| Renal Toxicity | 6 (13.0) | 2 (4.3) | 2 (4.1) | | 0 | | 8 (8.4) | 2 (2.1) |
| Embryo-fetal Toxicity* | 1 (2.2) | 0 | 1 (2.0) | | 0 | | 2 (2.1) | 0 |
| Impaired healing (wound) | 1 (2.2) | 0 | 0 | | 0 | | 1 (1.1) | 0 |
| Vascular disorders | 0 | 0 | 1 (2.0) | | 0 | | 1 (1.1) | 0 |

## Supplementary Table 8. Progression free survival based on local radiology review using the Kaplan-Meier method for NIS793 with spartalizumab and gemcitabine/nab-paclitaxel versus gemcitabine/nab-paclitaxel (Full Analysis Set).

NE=not estimable. $ Death before progression. # One-sided p-value obtained from unstratified log-rank test. ^Hazard ratio obtained from Cox PH model

|  | NIS793 with spartalizumab and gemcitabine/nab- paclitaxel N=50 |  | gemcitabine/nab- paclitaxel N=52 |
| --- | --- | --- | --- |
| Number of events-n (%) | 39(78.0) |  | 41(78.8) |
| with progression | 31(62.0) |  | 32(61.5) |
| with death $ | 8(16.0) |  | 9(17.3) |
| with censoring | 6(12.0) |  | 3(5.8) |
| P-value ARM1 versus ARM3 # |  | 0.46 |  |
| Hazard ratio (90% CI) ARM1 versus ARM3 ^ |  | 1.02 (0,1.37) |  |
| Kaplan-Meier estimate (%) (95% CI) |  |  |  |
| 3 months | 66.23 (50.34,78.09) |  | 69.97 (53.92,81.34) |
| 6 months | 36.40 (22.55,50.38) |  | 38.60 (24.13,52.88) |
| 12 months | 15.60 (6.47,28.35) |  | 18.19 (8.14,31.40) |
| 18 months | 10.40 (3.36,22.10) |  | 5.20 (0.95,15.30) |
| Percentiles (95% CI) |  |  |  |
| 25th | 2.07 (1.77,3.55) |  | 1.91 (0.79,3.68) |
| 50th | 3.91 (3.06,6.93) |  | 4.37 (3.55,7.20) |
| 75th | 7.36 (4.70,14.36) |  | 9.59 (5.49,14.95) |

## Supplementary Table 9. Progression free survival based on local radiology review using the Kaplan-Meier method for NIS793 with gemcitabine/nab-paclitaxel versus gemcitabine/nab-paclitaxel (Full Analysis Set).

NE=not estimable. $ Death before progression. # One-sided p-value obtained from unstratified log-rank test. ^Hazard ratio obtained from Cox PH model.

|  | NIS793 with gemcitabine/nab- paclitaxel N=51 |  | gemcitabine/nab- paclitaxel N=52 |
| --- | --- | --- | --- |
| Number of events-n (%) | 43(84.3) |  | 41(78.8) |
| with progression | 36(70.6) |  | 32(61.5) |
| with death $ | 7(13.7) |  | 9(17.3) |
| with censoring | 4(7.8) |  | 3(5.8) |
| P-value ARM2 versus ARM3 # |  | 0.38 |  |
| Hazard ratio (90% CI)ARM2 versus ARM3 ^ |  | 1.08 (0,1.44) |  |
| Kaplan-Meier estimate (%) (95% CI) |  |  |  |
| 3 months | 82.92 (68.74,91.07) |  | 69.97 (53.92,81.34) |
| 6 months | 42.68 (28.15,56.44) |  | 38.60 (24.13,52.88) |
| 12 months | 5.99 (1.28,16.23) |  | 18.19 (8.14,31.40) |
| 18 months | 3.00 (0.26,12.64) |  | 5.20 (0.95,15.30) |
| Percentiles (95% CI) |  |  |  |
| 25th | 3.71 (1.81,4.73) |  | 1.91 (0.79,3.68) |
| 50th | 5.52 (3.94,7.29) |  | 4.37 (3.55,7.20) |
| 75th | 8.11 (7.13,9.30) |  | 9.59 (5.49,14.95) |

## Supplementary Table 10. Analysis of overall survival using Kaplan-Meier method for NIS793 with spartalizumab and gemcitabine/nab-paclitaxel versus gemcitabine/nab-paclitaxel (Full Analysis Set)

|  | NIS793 with spartalizumab and gemcitabine/nab-paclitaxel N=50 | Gemcitabine/nab-paclitaxel N=52 |
| --- | --- | --- |
| Number of participants-n (%) |  |  |
| with death | 34 (68.0) | 40 (76.9) |
| with censorings | 16 (32.0) | 12 (23.1) |
| Censoring-free time (months), Percentiles (95% CI) |  |  |
| 25^th^ | 4.3 (2.6, 7.8) | 4.9 (2.6, 7.4) |
| 50^th^ | 10.7 (7.2, 12.7) | 10.1 (6.5, 13.4) |
| 75^th^ | 16.8 (11.8, 26.1) | 18.1 (12.2, 25.1) |
| KM estimates of being censored, (95% CI) |  |  |
| 3 months | 86.9 (73.1, 93.9) | 82.6 (68.2, 90.9) |
| 6 months | 72.9 (57.2, 83.7) | 71.7 (56.4, 82.5) |
| 12 months | 37.7 (23.2, 52.2) | 39.1 (25.2, 52.8) |
| 18 months | 24.4 (12.3, 38.6) | 26.1 (14.5, 39.2) |
| Unstratified log rank test |  |  |
| p-value (two sided) | 0.88 | |
| Cox proportional hazard ratio |  |  |
| Hazard ratio (95% CI) | 0.97 (0.61, 1.53) | |

## Supplementary Table 11. Analysis of overall survival using Kaplan-Meier method for NIS793 with gemcitabine/nab-paclitaxel versus gemcitabine/nab-paclitaxel (Full Analysis Set).

|  | NIS793 with gemcitabine/nab-paclitaxel N=51 | gemcitabine/nab-paclitaxel N=52 |
| --- | --- | --- |
| Number of participants-n (%) |  |  |
| with death | 41 (80.4) | 40 (76.9) |
| with censoring | 10 (19.6) | 12 (23.1) |
| Censoring-free time (months), Percentiles (95% CI) |  |  |
| 25^th^ | 6.4 (4.4, 7.9) | 4.9 (2.6, 7.4) |
| 50^th^ | 8.5 (7.7, 9.9) | 10.1 (6.5, 13.4) |
| 75^th^ | 12.3 (9.8, 17.6) | 18.1 (12.2, 25.1) |
| KM estimates of being censored, (95% CI) |  |  |
| 3 months | 91.7 (79.5, 96.8) | 82.6 (68.2, 90.9) |
| 6 months | 78.6 (63.9, 87.9) | 71.7 (56.4, 82.5) |
| 12 months | 29.2 (16.8, 42.8) | 39.1 (25.2, 52.8) |
| 18 months | 10.1 (3.2, 21.8) | 26.1 (14.5, 39.2) |
| Unstratified log rank test |  |  |
| p-value (two sided) | 0.22 | |
| Cox proportional hazard ratio |  | |
| Hazard ratio (95% CI) | 1.32 (0.84, 2.07) | |

**Supplementary Table 12. Pharmacokinetic analysis set for the NIS793, spartalizumab, gemcitabine and nab-paclitaxel as used in this study.**

*Data related to C1D15 and C3D15 pre-dose samples. n = number of participants with evaluable values, m = number of non-zero concentrations. Below the limit of quantitation (BLQ = 150 ng/mL) values have been set to zero. Zero concentrations are considered as missing in Geo-mean and Geo-CV% calculations.

| Parameter | Statistics | NIS793 with spartalizumab and gemcitabine/nab- paclitaxel | NIS793 with gemcitabine/nab- paclitaxel | Gemcitabine/nab- paclitaxel |  |
| --- | --- | --- | --- | --- | --- |
| NIS793 |  | **N=42** | **N=39** |  |  |
| Visit: Cycle 1 |  |  |  |  |  |
| AUC_last_ (h*ng/mL) | n | 42 | 39 |  |  |
|  | Geo-mean | 79300000 | 92600000 |  |  |
|  | Geo-CV% | 40.2 | 28.2 |  |  |
| C_max_ (ng/mL) | n | 42 | 39 |  |  |
|  | Geo-mean | 578000 | 590000 |  |  |
|  | Geo-CV% | 30.8 | 35.7 |  |  |
| T_max_ (h) | n | 42 | 39 |  |  |
|  | Median | 1.00 | 1.00 |  |  |
|  | Min-Max | 0.42-30.9 | 0.42-145 |  |  |
| Visit: Cycle 3 |  |  |  |  |  |
| AUC_last_ (h*ng/mL) | n | 21 | 31 |  |  |
|  | Geo-mean | 137000000 | 134000000 |  |  |
|  | Geo-CV% | 69.4 | 70.3 |  |  |
| C_max_ (ng/mL) | n | 21 | 31 |  |  |
|  | Geo-mean | 787000 | 717000 |  |  |
|  | Geo-CV% | 31.8 | 51.8 |  |  |
| T_max_ (h) | n | 21 | 31 |  |  |
|  | Median | 1.00 | 1.02 |  |  |
|  | Min-Max | 0.17-163 | 0-170 |  |  |
| Spartalizumab |  |  | N=44 |  |  |
| Visit: Cycle 1 |  |  |  |  |  |
| AUC_last_ (h*ug/mL) | n |  | 42 |  |  |
|  | Geo-mean |  | 9400 |  |  |
|  | Geo-CV% |  | 66.3 |  |  |
| C_max_ (ug/mL) | n |  | 42 |  |  |
|  | Geo-mean |  | 107 |  |  |
|  | Geo-CV% |  | 20.7 |  |  |
| T_max_ (h) | n |  | 42 |  |  |
|  | Median |  | 1.00 |  |  |
|  | Min-Max |  | 0.02-27.2 |  |  |
| Visit: Cycle 3 |  |  |  |  |  |
| AUC_last_ (h*ug/mL) | n |  | 21 |  |  |
|  | Geo-mean |  | 2460 |  |  |
|  | Geo-CV% |  | 144.3 |  |  |
| Gemcitabine |  | N=44 | N=43 | N=44 |  |
| Cycle 1 |  |  |  |  |  |
| AUC_last_ (h*ng/mL) | n | 42 | 40 | 44 |  |
|  | Geo-mean | 4760 | 6510 | 6240 |  |
|  | Geo-CV% | 155.9 | 251.4 | 118.0 |  |
| C_max_ (ng/mL) | n | 42 | 40 | 44 |  |
|  | Geo-mean | 6850 | 8700 | 8150 |  |
|  | Geo-CV% | 123.6 | 161.9 | 119.3 |  |
| T_max_ (h) | n | 42 | 40 | 44 |  |
|  | Median | 0.559 | 0.583 | 0.583 |  |
|  | Min-Max | 0.167-4.48 | 0.367-3.75 | 0.083-1.55 |  |
| Cycle 4 |  |  |  |  |  |
| AUC_last_ (h*ng/mL) | n | 23 | 28 | 24 |  |
|  | Geo-mean | 3120 | 4360 | 3670 |  |
|  | Geo-CV% | 207.6 | 114.3 | 145.8 |  |
| C_max_ (ng/mL) | n | 23 | 28 | 24 |  |
|  | Geo-mean | 5290 | 6530 | 5530 |  |
|  | Geo-CV% | 160.9 | 112.5 | 104.6 |  |
| T_max_ (h) | n | 23 | 28 | 24 |  |
|  | Median | 0.533 | 0.583 | 0.567 |  |
|  | Min-Max | 0.133-1.95 | 0.5-1.50 | 0.5-1.00 |  |
| Nab-paclitaxel |  | N=38 | N=39 | N=44 |  |
| Cycle 1 |  |  |  |  |  |
| AUC_last_ (h*ng/mL) | n | 37 | 38 | 43 |  |
|  | Geo-mean | 6190 | 4500 | 4150 |  |
|  | Geo-CV% | 125.8 | 51.7 | 65.0 |  |
| C_max_ (ng/mL) | n | 37 | 38 | 43 |  |
|  | Geo-mean | 3380 | 3100 | 2890 |  |
|  | Geo-CV% | 151.8 | 100.3 | 87.2 |  |
| T_max_ (h) | n | 37 | 38 | 43 |  |
|  | Median | 0.533 | 0.517 | 0.567 |  |
|  | Min-Max | 0.417-3.65 | 0.383-3.50 | 0.35-1.92 |  |
| Cycle 4 |  |  |  |  |  |
| AUC_last_ (h*ng/mL) | n | 19 | 28 | 24 |  |
|  | Geo-mean | 5060 | 4140 | 3670 |  |
|  | Geo-CV% | 63.5 | 81.4 | 59.5 |  |
| C_max_ (ng/mL) | n | 19 | 28 | 24 |  |
|  | Geo-mean | 3330 | 3250 | 2490 |  |
|  | Geo-CV% | 85.8 | 97.1 | 71.8 |  |
| T_max_ (h) | n | 19 | 28 | 24 |  |
|  | Median | 0.583 | 0.559 | 0.55 |  |
|  | Min-Max | 0-1.10 | 0.5-2.58 | 0.333-2.67 |  |

## Supplementary Table 13. Summary of preclinical toxicology findings.

| Species | Duration of dosing | Dose levels (mg/kg) | Major findings |
| --- | --- | --- | --- |
| Rat | 13-weeks  4-weeks of recovery | 0, 1, 3 and 10 IV | Liver: Increased liver weight at 3 and 10 mg/kg in M; minimal hypertrophy and increased hepatocyte vacuolation in 5/10 males each at 10mg/kg  Kidney: increased frequency in rat specific Chronic Progressive Nephropathy at 10 mg/kg compared to controls (10 mg/kg: M 9/10 and F 6/10. Control: M 5/10 and F 3/10)  Bone: Increase in number and thickness of bone trabeculae in femur at 10 mg/kg, M and F  Eyelids: Epidermal hyperplasia associated with mononuclear cell infiltrate of upper and lower eyelids in 5/10 M and 3/10 F in 10 mg/kg dose  All effects minimal to slight  NOAEL 10mg/kg |
| Cyno | 4-weeks  4-weeks of recovery | 0, 2, 8 and 16 IV | No toxicological or adverse findings including effects on CV, CNS and respiratory safety pharmacology were observed.  NOAEL 16mg/kg |
| Rat | 8 weeks | 0, 30, 90 every 10 days IV | Heart: minimal to moderate valvulopathy ≥30 mg/kg in 8/10 M at both doses  Cecum: minimal to slight mixed cell infiltration in 3/10 males at 90 mg/kg. |
| Cyno | 8 weeks | 0, 30/90 (on days 1, 11, 21 followed by 90 on days 31, 41, 51), 65 (every 10 days). IV | Cardiovascular system: at 65 mg/kg minimal to slight vascular inflammation/necrosis (heart, kidney, spleen), slight endothelial hyperplasia (heart) and slight myocardial inflammation in 1/3; at 65 mg/kg minimal angiectasis (pancreas) in 3/3.  Kidney: at 65 mg/kg slight tubulo-interstitial inflammatory processes (2/3), slight tubular hypertrophy/hyperplasia (2/3), slight inflammation renal pelvis (1/3)  Urinary bladder: at 65 mg/kg slight inflammation (1/3)  Stomach: At 65 mg/kg minimal increased mononuclear cell infiltrate (2/3)  Injection sites: At 30/90 mg/kg moderate hyperplasia endothelium (1/3), slight hypertrophy medial (1/3). At 30/90 and 65 mg/kg minimal to slight inflammation vascular (2/3 and 1/3), slight to moderate hemorrhage (2/3 and 1/3), minimal to slight edema (2/3 and 1/3) |
