# Supplementary material for: Biomarker Analysis from Patients with Metastatic PDAC Treated with TGFβ Antibody NIS793 plus Abraxane + Gemcitabine versus Abraxane + Gemcitabine Alone in a Phase II, Open-Label, Randomized Study
Source: Clin Cancer Res. 2026 Jun 25;32(18):4001–15. doi: 10.1158/1078-0432.CCR-26-0805 (PMC13575558; doi:10.1158/1078-0432.CCR-26-0805)
Supplement: Supplementary file 2 [file ccr-26-0805_supplementary_data2_suppds2.docx]

| Cancer types (s)/ subtypes(s)/ stage(s)/ condition | 1L Metastatic Pancreatic Adeno Carcinoma (PDAC) |
| --- | --- |
| Considerations related to: |  |
| Sex | Pancreatic ductal adenocarcinoma (PDAC) has a slightly higher incidence in males than in females globally, with an incidence of approximately 5.7 per 100,000 for males versus 4.1 per 100,000 for female. |
| Age | The median age of diagnosis for PDAC (PDAC) is 71 years old. While it is predominantly a disease of older adults—with about two-thirds of cases diagnosed in people 65 and older—nearly 10% to 15% of patients present with early-onset PDAC (under the age of 50 or 60). |
| Race/ethnicity | The highest rates of PDAC in the U.S. are reported in black people (approximately 15.9 per 100,000 people), White populations have a slightly lower incidence (roughly 12.6 per 100,000). Lower incidence rates reported in Hispanic and Asian/Pacific Islander individuals. |
| Geography | Highest rates of PDAC (7.9 to 9.9 per 100,000) occur in high-income regions like North America, Europe, Australia/New Zealand, and Japan. Rates are lowest (1.5 to 4.6 per 100,000) in Africa, Central America, and South Asia. |
| Overall representativeness of this study | Consistent with the slightly higher frequency of PDAC incidence in males, our study enrolled a slightly higher frequency of male patients (59.5% males versus 40.5% females). The age distribution of our study was similar to the average age distribution of PDAC in literature, with a median age of 66 for patients on study. Ethnicity distribution showed preferential enrollment of white individuals in this study (white, 69.9%; black, 3.3%; Asian, 25.49%). |

**Supplementary Table: Representativeness of study participants**
